# Negative Regulation of TLR Inflammatory Signaling by the SUMO-deconjugating Enzyme SENP6

**DOI:** 10.1371/journal.ppat.1003480

**Published:** 2013-06-27

**Authors:** Xing Liu, Wei Chen, Qiang Wang, Li Li, Chen Wang

**Affiliations:** State Key Laboratory of Cell Biology, Institute of Biochemistry and Cell Biology, Shanghai Institutes for Biological Sciences, Chinese Academy of Sciences, Shanghai, China; Harvard Medical School, United States of America

## Abstract

The signaling of Toll-like receptors (TLRs) induces host defense against microbial invasion. Protein posttranslational modifications dynamically shape the strength and duration of the signaling pathways. It is intriguing to explore whether de-SUMOylation could modulate the TLR signaling. Here we identified SUMO-specific protease 6 (SENP6) as an intrinsic attenuator of the TLR-triggered inflammation. Depletion of SENP6 significantly potentiated the NF-κB-mediated induction of the proinflammatory genes. Consistently, SENP6-knockdown mice were more susceptible to endotoxin-induced sepsis. Mechanistically, the small ubiquitin-like modifier 2/3 (SUMO-2/3) is conjugated onto the Lysine residue 277 of NF-κB essential modifier (NEMO/IKKγ), and this impairs the deubiquitinase CYLD to bind NEMO, thus strengthening the inhibitor of κB kinase (IKK) activation. SENP6 reverses this process by catalyzing the de-SUMOylation of NEMO. Our study highlights the essential function of the SENP family in dampening TLR signaling and inflammation.

## Author Summary

Given the double-edged nature of the TLR functions, the strength and duration of the TLR signaling are dynamically modulated via protein posttranslational modifications, which ensure that the invading microbes are quickly eliminated and the damages to the host are reduced to the minimum. Besides the ubiquitin, ubiquitin-like proteins are emerging as novel protein tags to fine-tune the TLR signaling. It is intriguing to explore how the SUMO-specific proteases (SENPs) contributes to balancing the SUMOylation status of the TLR signaling. Our study reveals that NEMO, a critical protein of the TLR signaling pathways, is covalently modified by SUMO-2/3. This modification is resversed by the de-SUMOylation activity of SENP6. Thus, SENP6 faciliates CYLD to bind NEMO and to remove the polyubiquitin chains on NEMO, ultimately dampening the IKK activation. This study sheds new light on the dynamic functions of the SUMOylation in restricting proinflammatory response.

## Introduction

Toll like receptors (TLRs) are a family of membrane receptors that sense a wide range of invading pathogens, including bacteria, fungi and viruses. Upon activation, TLRs trigger innate immune responses and prime the adaptive immune system to eliminate the pathogens [1], [2]. However, the excessive activation of TLR signaling causes injuries to the host (inflammation and autoimmune diseases) [3]. Thus, the TLR signaling pathways are subjected to stringent regulations spatially and temporally.

TLR signaling triggers the activation of NF-κB, interferon-regulatory factors (IRFs) and activator protein 1 (AP-1). These transcriptional factors coordinate to induce the expression of a broad range of proteins important in the immune and inflammatory responses [4], [5]. TLR-mediated activation of NF-κB depends on the activity of the inhibitor of NF-κB (IκB) kinase (IKK) complex. The IKK complex is composed of two related catalytic subunits, IKKα and IKKβ, and a regulatory subunit, NF-κB essential modifier (NEMO/IKKγ) [6], [7]. Although NEMO does not display catalytic activity, it is indispensable for the activation of the IKKα/β [8], [9]. Recent studies propose that NEMO contains the unique ubiquitin-binding domain, which recognizes the K63-linked and linear polyubiquitin chains and triggers IKK activation [10], [11]. Intriguingly, NEMO *per se* is modified by the polyubiquitin chain, which is also critical for the IKK activation [12], [13]. Notably, the deubiquitinase CYLD could interact with NEMO and cleave these polyubiquitin chains, thus acting as a negative regulator of NF-κB signaling [14].

Interestingly, NEMO and IκBα are dynamically modified by SUMO-1 [15], [16]. The SUMO-1 modification of IκBα makes it resistant to the signal-induced degradation. It is intriguing to understand the potential function of the SUMOylation of NEMO, in particular, to address the synergistic or antagonistic effect between the ubiquitination and SUMOylation of NEMO. Futhermore, it remains unknown whether NEMO could be modified by SUMO-2/3 [17].

Like ubiquitination, SUMOylation is a dynamic process, which involves three classes of enzymes: E1 activating enzyme (SAE1/SAE2), E2 conjugating enzyme (Ubc9) and possibly E3 ligases. SUMOylation is reversed by a family of sentrin/SUMO-specific proteases (SENPs) [18], [19]. SENP family has six members (SENP1-3 & SENP5-7), each of which exhibits distinct expression patterns and substrate specificity [20], [21].

Much is known about the biological functions of SENP1 and SENP2. For example, SENP1 and SENP2 could process newly synthesized SUMOs into their mature forms. SENP1 plays critical roles in the hypoxic responses, by reversing the SUMOylation of HIF1α and impairing the VHL protein to bind HIF1α, thus stabilizing HIF1α [22]. SENP2 modulates adipogenesis by the de-SUMOylation and stabilization of C/EBPβ [23]. SENP2 is essential for suppressing the polycomb group protein-mediated gene silencing, via targeting Pc2/CBX4, during embryonic development [24]. However, the physiological functions of the other SENPs are largely unknown, and this represents an emerging frontier for further investigation.

In this study, we report that SUMO-2/3 are conjugated onto the Lysine residue 277 of NEMO. This modification prevents the deubiquitinase CYLD from binding to NEMO and thus strengthens the IKK activation. SENP6 specifically reverses this process by catalyzing the de-SUMOylation of NEMO. Knockdown of SENP6 significantly potentiates the TLR-mediated induction of the proinflammatory genes. The *in vivo* ‘knockdown’ of SENP6 by siRNA confirms its critical role in tolerance to LPS in endotoxic shock models. This study reveals the essential function of SENP6 for dampening TLR-induced inflammation, shedding new light on the dynamic functions of the SUMOylation in innate immunity.

## Results

### Identification of SENP6 as a novel regulator of TLR signaling

**Figure 1:**
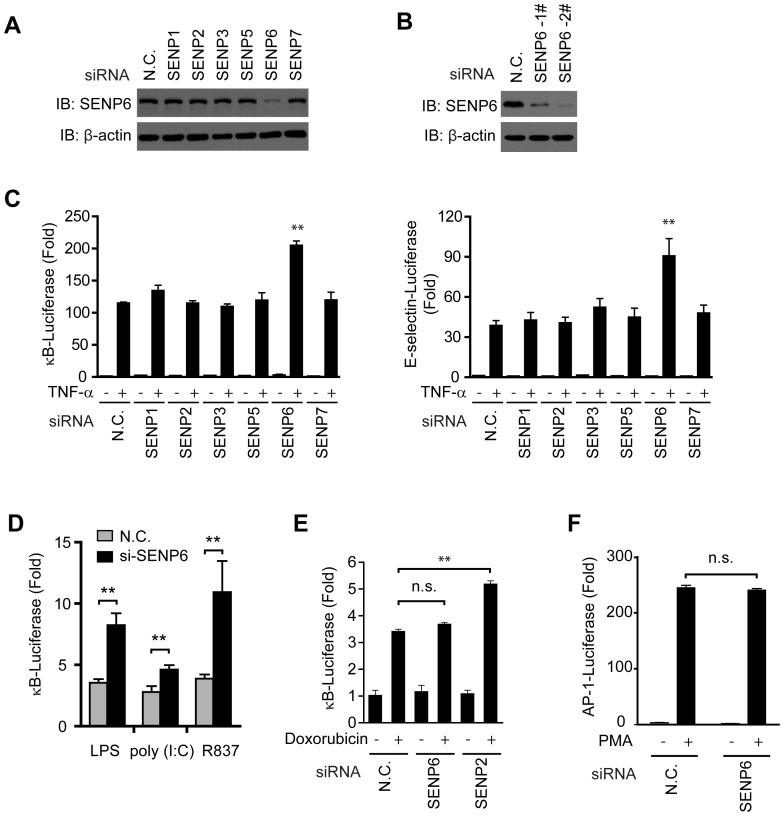
Identification of SENP6 as a new regulator of TLR signaling. A, Immunoblot analysis of the lysates from 293T cells transfected with the indicated siRNA. B, Immunoblot analysis of the lysates from MEF cells transfected with the indicated siRNA. C, SENP6 potentiates TNF-α-induced NF-κB activation. 5×κB-luciferase (left panel) or E-selectin-luciferase (right panel) and pTK-Renilla reporters were transfected into HEK293T cells together with the indicated siRNA. Forty-eight hours after transfection, cells were stimulated with TNF-α (10 ng/ml) for eight hours before luciferase reporter assays were performed. D, SENP6 potentiates TLR-triggered NF-κB activation. The nonspecific control (N.C.) or SENP6 siRNA were transfected into RAW264.7 cells with 5×κB-luciferase and pTK-Renilla reporters. Forty-eight hours after transfection, cells were stimulated with LPS (1 µg/mL), poly (I:C) (50 µg/ml), R837 (10 µg/ml) for 8 h before luciferase assays were performed. E, SENP6 has no effect on the NF-κB activation in response to DNA damage. HEK293T cells were transfected with 5×κB-luciferase and pTK-Renilla reporters together with the nonspecific control (N.C.), SENP6 siRNA or SENP2 siRNA. Forty-eight hours after transfection, cells were stimulated with doxorubicin (500 ng/ml) for eight hours before luciferase reporter assays were performed. F, SENP6 has no influence on the activation of AP-1. HEK293T cells were transfected with AP-1-luc and pTK-Renilla reporters together with the nonspecific control (N.C.) or SENP6 siRNA. Forty-eight hours after transfection, cells were stimulated with PMA (20 ng/ml) for eight hours before luciferase reporter assays were performed. Data from C-F are presented as means ±S.D. from three independent experiments. n.s., not significant; *, P<0.05; **, P<0.01.

To probe the potential function of the SUMO-specific proteases, we screened out individual siRNAs, which respectively diminish the expression of the corresponding SENPs (Fig. 1A, 1B and Fig. S1). An RNAi-based screening showed that the depletion of SENP6, rather than other members of the SENP family, apparently enhanced the TNF-α-induced activation of the κB-luciferase reporter and the E-selectin-luciferase reporter, which are both tightly regulated by NF-κB (Fig. 1C). Likewise, the activation of κB-luciferase reporter was apparently boosted upon SENP6 depletion in response to TLR4, TLR3 or TLR7 signaling in RAW264.7 cells when stimulated with extracellular LPS, poly (I:C), imiquimod (R837), respectively (Fig. 1D).

A recent study suggested that SENP2 could potentially inhibit the NF-κB activation in response to DNA damage [25], which is confirmed by us (Fig. 1E). However, silencing of SENP6 apparently displayed no effect on the genotoxic-stress-induced NF-κB activation (Fig. 1E). Neither could SENP6 influence the PMA-induced activation of the AP-1-luciferase reporter (Fig. 1F). These data suggest that SENP6 may play a negative regulatory role for TLR signaling.

### Knockdown of SENP6 potentiates TLR-mediated NF-κB activation

**Figure 2:**
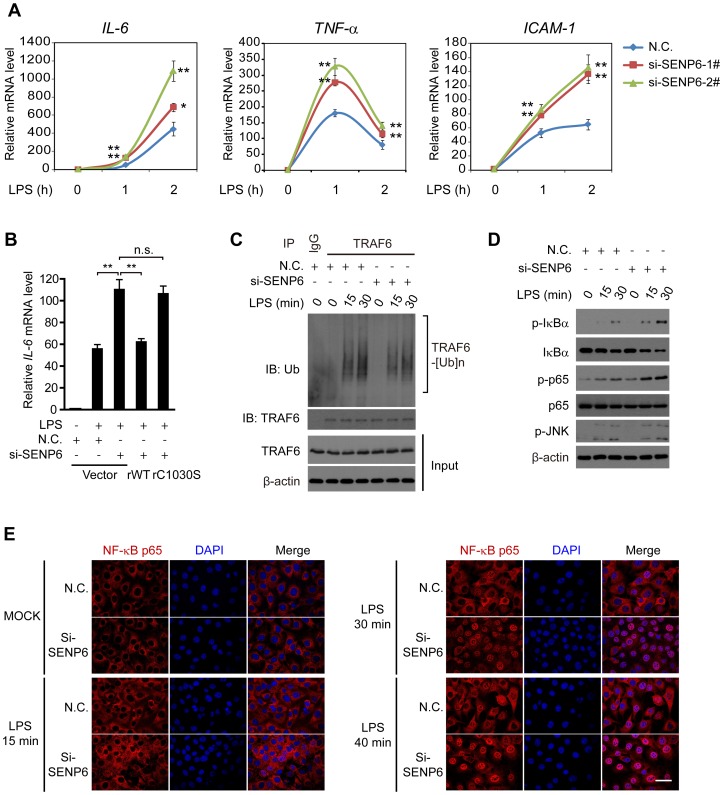
SENP6 negatively regulates TLR4-induced NF-κB signaling. A, Loss of SENP6 promotes LPS-induced NF-κB-responsive genes. MEF cells transfected with the indicated siRNAs were incubated with LPS (1 µg/mL) for the indicated time periods. Induction of *IL-6*, *TNF-α*, and *ICAM-1* mRNA was measured by quantitative PCR. B, Catalytic activity of SENP6 is required for its action in TLR signaling. MEF cells were transfected with the nonspecific control (N.C.) or SENP6 siRNA and then rescued with the indicated siRNA-resistant SENP6 constructs. After LPS (1 µg/mL) stimulation, induction of *IL-6* mRNA was measured by quantitative PCR. C, SENP6 has no effects on LPS-induced auto-ubiquitination of TRAF6. Immunoprecipitation of endogenous TRAF6 from lysates of LPS-treated MEF cells transfected with the nonspecific control (N.C.) or SENP6 siRNA, followed by immunoblot analysis with the indicated antibodies. D, SENP6 knockdown enhances the phosphorylation of IκBα and NF-κB p65. Immunoblot analysis of phosphorylated (p-) IκBα, NF-κB p65 and JNK in WT and SENP6 knockdown MEFs stimulated with LPS (1 µg/mL). E, RNAi of SENP6 accelerates the nuclear translocation of p65. Immunofluorescence microscopy of NF-κB p65 (red) in WT and SENP6 knockdown MEF cells stimulated with LPS (1 µg/mL); nuclei were counterstained with DAPI (blue). Scale bar, 50 µm. Original magnification, ×63. Data in A–B are presented as means ± S.D. from three independent experiments. *, P<0.05; **, P<0.01.

To substantiate, we explored the effect of SENP6 knockdown on the expression of the endogenous NF-κB-responsive genes induced by LPS, using qPCR (quantitative PCR) and ELISA (enzyme-linked immunosorbent assay). As expected, SENP6 knockdown markedly potentiated the induction of the NF-κB-responsive genes (*IL-6*, *TNF-α*, and *ICAM-1*), whereas SENP7 knockdown displayed no such effect (Fig. 2A, Fig. S2 and Fig. S3). In contrast, the induction of IRF3-responsive genes (*ISG15* and *ISG56*) was unaffected by SENP6 depletion upon LPS stimulation (Fig. S4). Consistently, in response to poly (I:C) or Sendai virus stimulation, knockdown of SENP6 resulted in augmented production of NF-κB-targeted cytokines as well (Fig. S3 and Fig. S5).

To rule out the potential off-target effects of the SENP6 siRNA, we generated several RNA interference (RNAi)-resistant SENP6 constructs, namely rSENP6 WT and rSENP6 C1030S (the catalytically inactive mutant which loses the de-SUMOylation activity, see below), in which silent mutations were introduced into the sequence targeted by the siRNA without changing the amino acid sequence of the corresponding proteins. MEF cells were first transfected with control or SENP6 siRNAs, followed by transfection of the control or indicated rSENP6 plasmids, respectively. Then, the induction of *IL-6* mRNA was measured after LPS stimulation. As shown in Fig. 2B, the up-regulation of the *IL-6* mRNA, in SENP6 knockdown cells, was reversed by introducing rSENP6 WT. But this is not reversed by rSENP6 C1030S. Collectively, these data indicate that SENP6 negatively modulates TLR-mediated NF-κB signaling, and this is dependent on its de-SUMOylation activity.

To further elucidate the signaling node targeted by SENP6, we observed that LPS-induced TRAF6 auto-ubiquitination was not affected by endogenous SENP6 depletion (Fig. 2C). In contrast, knockdown of SENP6 led to an apparent increase in the phosphorylation of IκBα and NF-κB p65, but not that of JNK (Fig. 2D). Consistently, the nuclear translocation of p65, induced by LPS, was markedly accelerated when depleting the SENP6 (Fig. 2E). In addition, we observed that exogenous expression of MyD88, TRAF6 or IKKβ could respectively activate the κB-luciferase reporters, and these activations were markedly potentiated when knocking down SENP6 (Fig. S6). In contrast, SENP6 knockdown had no effect on the activation of the κB-luciferase reporter, when cells were stimulated with the exogenous p65 (Fig. S6). Given the hierarchical relationships among these signaling molecules, we reasoned that SENP6 modulates NF-κB activation around the IKK protein complex.

### NEMO/IKKγ is specifically modified by SUMO-2/3

**Figure 3:**
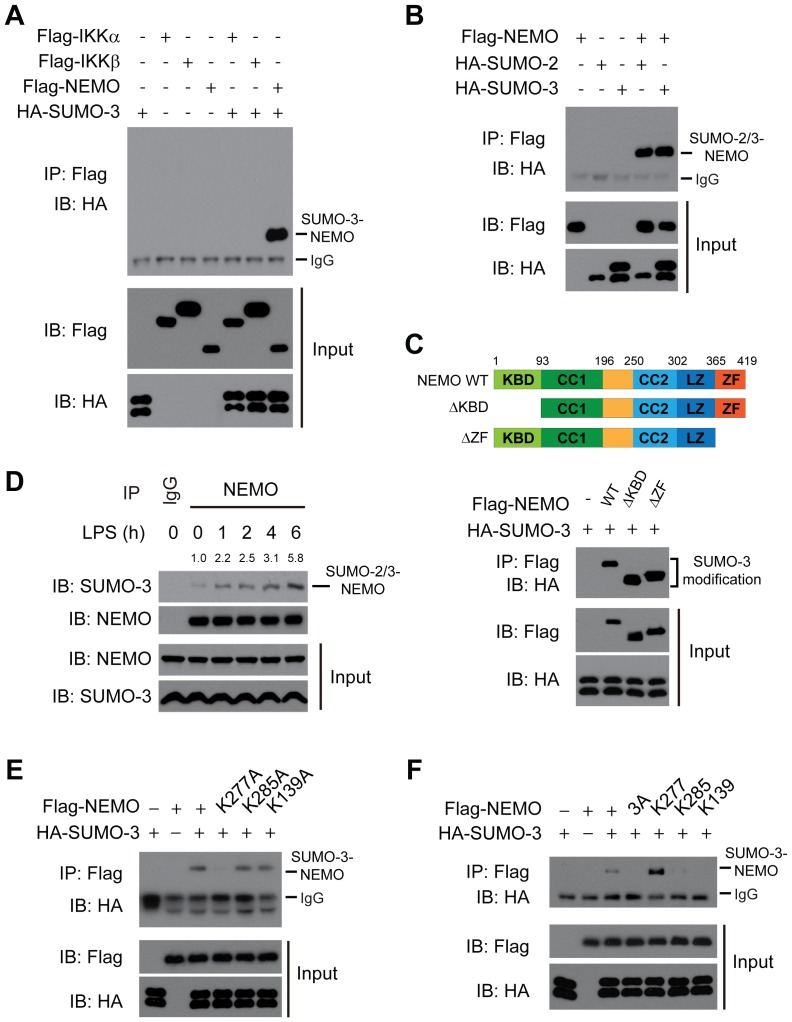
NEMO is modified on K277 by SUMO-2/3. A, NEMO, rather than IKKα or IKKβ, is modified by SUMO-3 in an overexpression system. Flag-IKKα, IKKβ or NEMO were individually transfected into HEK293T cells along with HA-SUMO-3. Cell lysates were subjected to denaturing immunoprecipitation with Flag antibody and then immunoblotted with the indicated antibodies. B, Either SUMO-2 or SUMO-3 could be attached to NEMO. Flag-NEMO was co-transfected into HEK293T along with HA-SUMO-2 or HA-SUMO-3. Cell lysates were subjected to denaturing immunoprecipitation with Flag antibody and then immunoblotted with the indicated antibodies. C, The SUMOylation of NEMO is independent of its IKK-binding domain. Expression vectors for Flag-NEMO and its mutants as shown in upper panel were transfected into 293T cells along with HA-SUMO-3 (lower panel). Cell lysates were subjected to immunoprecipitation with Flag antibody and then immunoblotted with the indicated antibodies. KBD, IKK-binding domain; CC1 and CC2, coiled-coil domain 1 and 2; LZ, leucine-zipper motif; ZF, zinc-finger domain. D, Endogenous NEMO is covalently modified by endogenous SUMO-2/3. After mock or LPS (1 µg/ml) stimulation, lysates from RAW264.7 cells were immunoprecipitated with NEMO antibody or control IgG and then immunoblotted with the indicated antibodies. The intensity of the SUMOylated NEMO was quantified and normalized to that of the corresponding immunoprecipitated NEMO. The relative levels of SUMOylated NEMO are shown as fold change compared with the control. E, K277 was the major acceptor site on NEMO for SUMO-3. HEK293T cells were transfected with Flag-NEMO or its mutants along with HA-SUMO-3. Cell lysates were subjected to immunoprecipitation with Flag antibody and then immunoblotted with the indicated antibodies. F, K277 of NEMO is sufficient for the SUMO-3 modification. HEK293T cells were transfected with the indicated plasmids. Cell lysates were subjected to immunoprecipitation with Flag antibody and then immunoblotted with the indicated antibodies.

Biochemically, SENP6 has been characterized to preferentially remove SUMO-2/3 from the pseudo-substrate [20]. Given the observation that SENP6 modulates the NF-κB signaling via its de-SUMOylation activity, we hypothesized that the IKK complex is a target for the SUMO-2/3 modification. To explore this possibility, IKKα, IKKβ or NEMO was respectively co-expressed with SUMO-3 in HEK293T cells. The cell lysates were subjected to the denaturing immunoprecipitation of Flag (Fig. 3A) or the Ni-NTA pulldown of His-SUMO-3 (Fig. S7A). Then the precipitates were probed with the indicated antibodies. Apparently, NEMO was robustly modified by SUMO-3, whereas neither IKKα nor IKKβ could be modified by SUMO-3 (Fig. 3A and Fig. S7A).

Sequence alignment reveals that SUMO-2 and SUMO-3 are almost identical (∼95% identical), whereas SUMO-1 is largely diverged (∼45% identical) [20]. Indeed, besides SUMO-3, SUMO-2 could be conjugated onto NEMO as well (Fig. 3B and Fig. S7B). Additionally, this modification took place in a IKKα and IKKβ-independent manner, as deletion of the N-terminal IKK-binding domain of NEMO [26] did not affect the SUMOylation of NEMO (Fig. 3C). Furthermore, endogenous NEMO was confirmed to be SUMOylated as the above observations (Fig. 3D). Interestingly, the SUMOylation of NEMO was markedly enhanced when stimulated respectively with LPS (TLR4 agonist), poly (I:C) (TLR3 agonist), or imiquimod R837 (TLR7 agonist) (Fig. 3D and Fig. S8). Taken together, these data indicated that exogenously expressed and endogenous NEMO can be targeted for SUMO-2/3 modification.

A highly conserved motif ΨKxD/E (where Ψ is a hydrophobic residue and x represents any residue) has been proposed as the SUMOylation sites for any given substrates [19]. We uncovered three putative SUMO conjugation sites on NEMO(K139, 277 and 285) through bioinformatics analysis. Then, we carried out a systematic lysine (K) to alanine (A) mutation scanning to identify the potential SUMOylation sites on NEMO. SUMO-3 was expressed along with the NEMO constructs harboring different K to A point mutations, followed by IP or Ni-NTA pulldown analysis. Whereas NEMO K139A and NEMO K285A were SUMOylated as well as the wild-type NEMO, the SUMOylation of the NEMO K277A was almost abolished (Fig. 3E and Fig. S7C), indicating that the K277 was the major acceptor site on NEMO for SUMO-3.

Alternatively, we generated the NEMO (3A) mutant, in which all of the three lysines (139, 277 and 285) were mutated to alanines. As expected, NEMO (3A) could barely be modified by SUMO-3 (Fig. 3F and Fig. S7D). On the background of this NEMO (3A) mutant, we generated three more NEMO mutants (K139, K277 or K285 respectively), in which a lysine residue was re-introduced back to the original site. We observed that, only when K277 was re-installed into the NEMO (3A) mutant could the SUMOylated band re-appeared (Fig. 3F and Fig. S7D). Collectively, these data firmly establish that the K277 of NEMO is both necessary and sufficient for the SUMO-3 modification.

### SENP6 catalyzes the de-SUMOylation on the NEMO K277

Given that NEMO could be modified by SUMO-2/3, we speculated that the SUMOylated NEMO is a potential substrate of SENP6. To address this possibility, we analysed the catalytic center of the SENP family (Fig. S9A) and generated several SENP6 point mutants. The SENP6 C1030S (Cys to Ser mutation at 1030 residue) is the catalytically inactive mutant [27]; whereas the SENP6 D1029E (Asp to Glu mutation at 1029 residue) is the catalytically active one, serving as a control.

**Figure 4:**
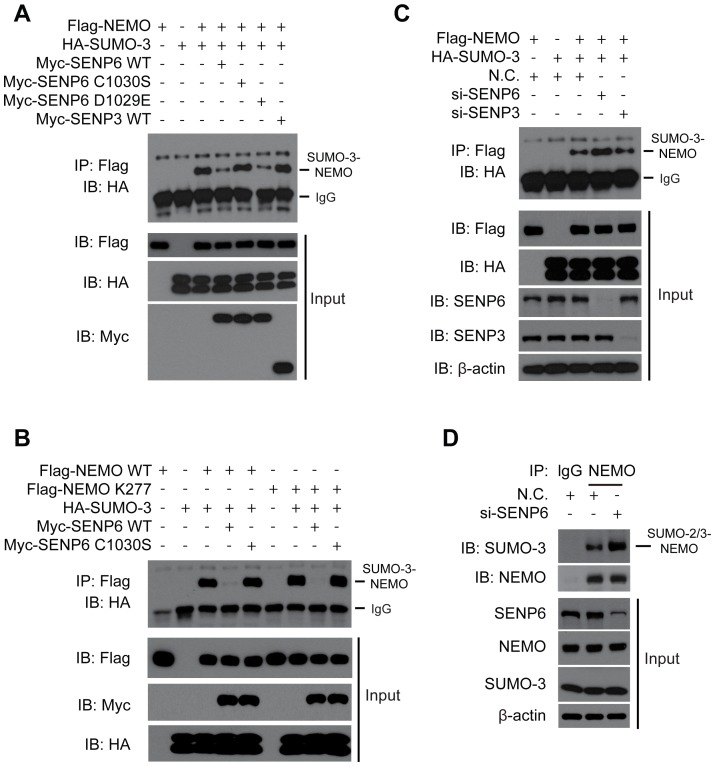
SENP6 catalyzes the de-SUMOylation on NEMO K277. A, Overexpression of catalytic active SENP6 deconjugates SUMOylated NEMO. Flag-NEMO and HA-SUMO-3 were transfected into HEK293T cells along with Myc tagged SENP6, SENP6 mutants or SENP3, respectively. 36 hours post-transfection, cell lysates were immunoprecipitated with Flag antibody and then immunoblotted with the indicated antibodies. B, SENP6 removes SUMO-3 attached on NEMO K277. HEK293T cells were transfected with the indicated plasmids. 48 hours post-transfection, cell lysates were subjected to immunoprecipitation with Flag antibody and then immunoblotted with the indicated antibodies. C, SENP6 knockdown enhance SUMOylation of exogenous NEMO. HEK293T cells were transfected with the indicated siRNAs. Twenty-four hours later, Flag-NEMO and HA-SUMO-3 were transfected into the knockdown cells. Cell lysates were subjected to immunoprecipitation with Flag antibody and immunoblotted with the indicated antibodies. D, The level of modified endogenous NEMO by SUMO-2/3 is elevated upon SENP6 depletion. HEK293T cells were transfected with the indicated siRNAs. Forty-eight hours later, cell lysates were subjected to immunoprecipitation with NEMO antibody or control IgG and then immunoblotted with the indicated antibodies.

A cell-based de-SUMOylation assay was performed to determine whether SENP6 could deconjugate the SUMOylated NEMO. The indicated SENP6, SENP3 or SENP7 construct was individually co-transfected with NEMO and SUMO-3. The cell lysates were subjected to immunoprecipitation of Flag-NEMO (Fig. 4A and Fig. S10) or Ni-NTA pulldown of His-SUMO-3 (Fig. S9B). Then the precipitates were probed with the indicated antibodies. As expected, NEMO was robustly SUMOylated in the presence of SUMO-3. Notably, this modification was drastically reduced by the expression of SENP6. In contrast, SENP6 C1030S, SENP3 as well as SENP7 could not influence the SUMOylation status of NEMO. In addition, SENP6 D1029E decreased the NEMO SUMOylation similar to that of the SENP6 (WT) (Fig. 4A and Fig. S9B).

We went on to investigate whether SENP6 can remove the SUMO-3 modification on the NEMO K277. As expected, the NEMO K277 (see above) was modified by SUMO-3, as well as that of the NEMO (WT). Notably, the conjugation of SUMO-3 onto the K277 of NEMO was almost completely abolished in the presence of SENP6 (WT). In contrast, SENP6 C1030S could not influence the SUMOylation status of the NEMO K277 (Fig. 4B). Furthermore, knocking down endogenous SENP6 could enhance the basal SUMOylation of Flag-NEMO, whereas knocking down endogenous SENP3 displayed no such effect (Fig. 4C). Consistently, SENP6 knockdown apparently potentiated the SUMOylation of the endogenous NEMO (Fig. 4D). Collectively, these data demonstrate that SENP6 deconjugates the SUMO-3 modification on the K277 of NEMO.

### SENP6 selectively interacts with the SUMOylated NEMO

**Figure 5:**
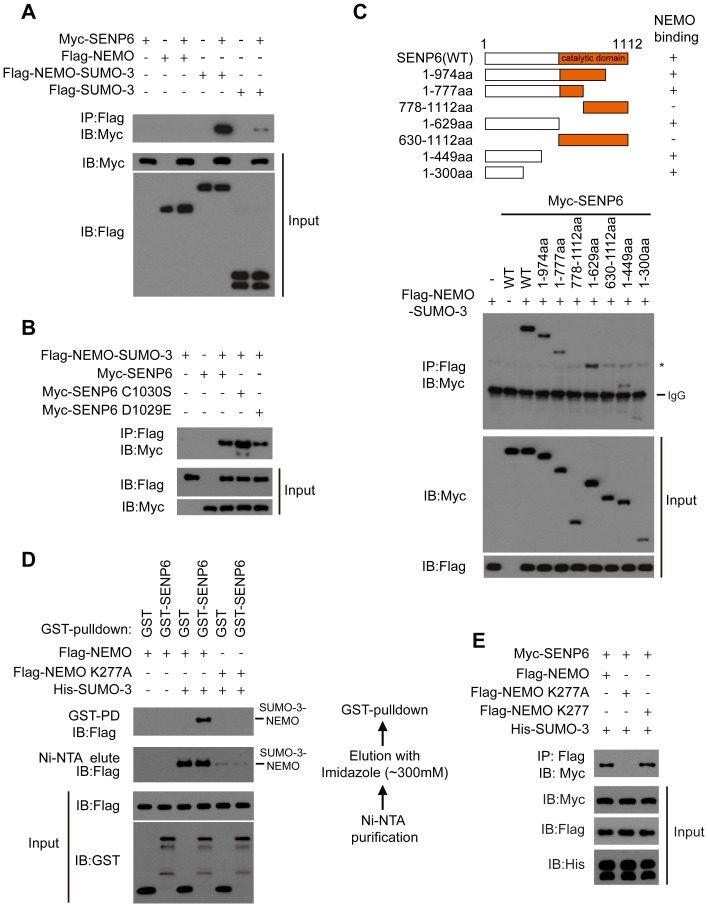
SENP6 selectively interacts with the SUMOylated NEMO. A, SENP6 associates with the NEMO-SUMO-3 fusion protein. The indicated Flag tagged constructs were individually transfected into HEK293T cells along with Myc-SENP6. Then, cell lysates were subjected to immunoprecipitation with Flag antibody. The immunoprecipitates were immunoblotted with the indicated antibodies. B, The catalytic activity of SENP6 is not required for the association of SENP6 and NEMO-SUMO-3. HEK293T cells were transfected with Flag-NEMO-SUMO-3 along with Myc-SENP6 or SENP6 mutants. Cell lysates were subjected to immunoprecipitation with Flag antibody. The immunoprecipitates were immunoblotted with the indicated antibodies. C, Mapping the binding domain of SENP6 with NEMO-SUMO-3. Myc-SENP6 and its mutants, as shown in upper panel, were individually transfected into HEK293T cells along with Flag-NEMO-SUMO-3. The cell lysates were immunoprecipitated with Flag antibody and then immunoblotted with the indicated antibodies (lower panel). A nonspecific band is indicated by an asterisk. D, SENP6 interacts with the SUMOylated NEMO. The flow chart of the GST-pulldown analysis (right panel). HEK293T cells were transfected with the indicated plasmids. Firstly, the cell extracts were purified by Ni-NTA agarose. Then the Ni-NTA-bound proteins were eluted by imidazole (∼300 mM), and the elute fractions were subjected to GST-PD analysis. The SUMOylated Flag-NEMO was indicated on the right (left panel). E, Flag-NEMO and its mutants were individually transfected into HEK293T cells along with His-SUMO-3, and then the cell lysates were incubated with the extracts from the HEK293T cells transfected with Myc tagged SENP6. The mixture was subjected to immunoprecipitation with Flag antibody and then immunoblotted with the indicated antibodies.

We noticed that SENP6 could not co-immunoprecipitate with NEMO *per se*. However, SENP6 could co-immunoprecipitate weakly with SUMO-3 alone (Fig. 5A). This behavior is similar to many ubiquitin-like binding proteins, which bind to the ubiquitin-like fusion proteins (mimicking the constitutively modified state of a given substrate). So we generated the NEMO-SUMO-3 fusion protein, in which SUMO-3 was fused to the C-termini of NEMO. As shown in Fig. 5A, SENP6 interacts strongly with the NEMO-SUMO-3, as compared to SUMO-3 or NEMO alone. Furthermore, we explored the interaction between SENP6 mutants and the NEMO-SUMO-3. It was observed that NEMO-SUMO-3 could interact as well with SENP6 C1030S or SENP6 D1029E (Fig. 5B). Notably, SENP6 C1030S displayed substantially improved binding affinity for NEMO-SUMO-3 (Fig. 5B), suggesting that the enzymatic activity of SENP6 is dispensable for its binding.

To map the critical domain for this interaction, a series of Myc tagged SENP6 deletion mutants (Fig. 5C, upper panel) were generated and individually transfected into HEK293T cells along with the NEMO-SUMO-3. It was observed that the N terminal domain of SENP6 (1–300 aa) mediated this interaction (Fig. 5C, lower panel).

To confirm that SENP6 does interact with the SUMOylated NEMO, the SUMOylated NEMO was generated and purified as shown in the experimental procedure (Fig. 5D, right panel). Briefly, Flag-NEMO was co-expressed with His-SUMO-3 and the cell extracts were purified by Ni-NTA agarose and the eluate (∼300 mM Imidazole) was subjected to GST-pulldown. Consistently, the GST-SENP6 could directly pull down the SUMOylated NEMO, whereas GST failed to do so. In addition, GST-SENP6 could not pull down anything from the NEMO K277A eluate (Fig. 5D, left panel). Consistently, SENP6 was co-immunoprecipitated by either NEMO or NEMO K277 in the presence of SUMO-3 (Fig. 5E). However, this does not apply to SENP7 (Fig. S11). Taken together, these data indicate that SENP6 could specifically interact with the SUMOylated NEMO, but not with NEMO *per se*.

### SENP6 attenuates the action of NEMO in TLR-triggered NF-κB signaling

**Figure 6:**
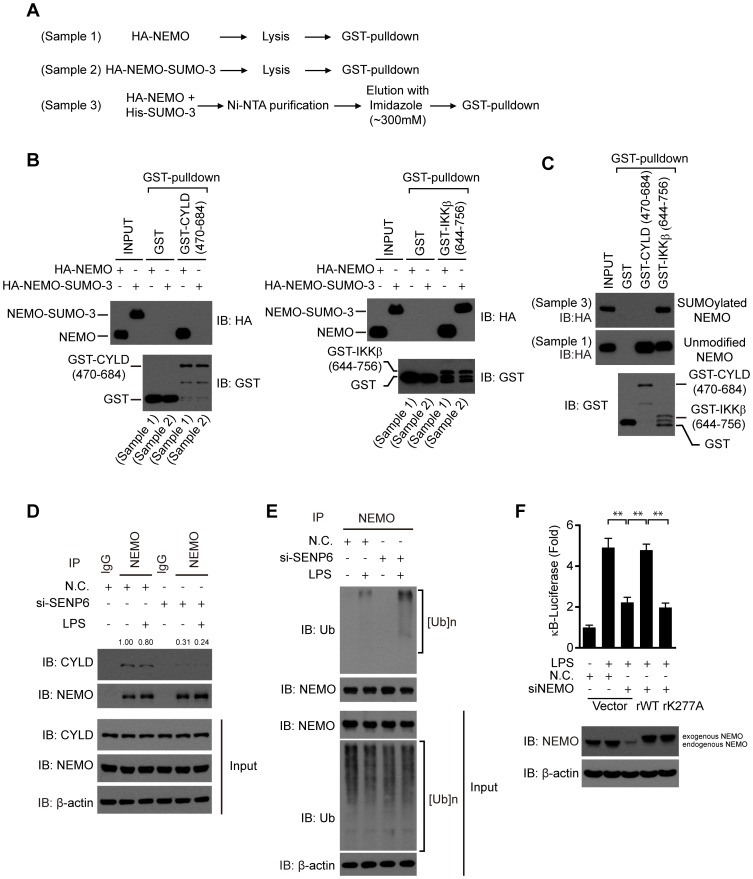
SENP6 attenuates the action of NEMO in TLR-triggered NF-κB signaling. A, The flow charts of the GST-pulldown analysis. HEK293T cells were transfected with the indicated plasmids. When HA-NEMO (Sample 1) or HA-NEMO-SUMO-3 (Sample 2) was expressed alone, the cell extracts were subjected directly to GST-PD analysis. When HA-NEMO was co-expressed with His-SUMO-3, the cell extracts were firstly purified by Ni-NTA agarose. Then the Ni-NTA-bound His-SUMO-3 modified proteins were eluted by imidazole (∼300 mM), and the elute fractions were subjected to GST-PD analysis (Sample 3). B, HEK293T cells were transfected with HA tagged NEMO or NEMO-SUMO-3, and then equal amounts of cell lysates were subjected to GST-PD as indicated according to the experimental design described in A. C, HEK293T cells were transfected with HA-NEMO together with His-SUMO-3. The His-SUMO-3 modified proteins purified from the cell extracts were analyzed by GST-PD analysis as indicated according to the experimental design described in A. The SUMOylated and unmodified HA-NEMO were indicated on the right. D, SENP6 depletion abrogates the endogenous interaction between NEMO and CYLD. After mock or LPS (1 µg/ml) stimulation, lysates from MEF cells were immunoprecipitated with anti-NEMO antibody or anti-IgG and then immunoblotted with an anti-CYLD antibody. The intensity of the CYLD bound to NEMO was quantified and normalized to that of the corresponding immunoprecipitated NEMO. The relative levels of co-immunoprecipitated CYLD are shown as fold change compared with the control. E, SENP6 inhibits LPS-induced attachment of polyubiquitin chains onto NEMO. MEF cells were transfected with the nonspecific control (N.C.) or SENP6 siRNA and then treated with LPS (1 µg/mL). Cell lysates were immunoprecipitated with anti-NEMO and analyzed by immunoblotting using anti-ubiquitin (Ub). F, Modification of NEMO by SUMO-3 is required for the activation of NF-κB. RAW264.7 cells were transfected with the nonspecific control (N.C.) or NEMO siRNA together with 5×κB-luciferase and pTK-Renilla reporters, as well as the indicated siRNA-resistant NEMO constructs. After LPS (1 µg/ml) stimulation, cell lysates were prepared for luciferase assay. Data from F are presented as means ± S.D. from three independent experiments. *, P<0.05; **, P<0.01.

To explore the functional consequence of the SUMOylation of NEMO, we tested whether this modification will influence the composition of the NEMO protein complex. So, the SUMOylated NEMO or the NEMO-SUMO-3 fusion protein was produced and purified, and their interactions with other regulatory proteins were investigated (Fig. 6A). Consistent with recent reports [14], [26], both GST-CYLD (470–684 aa) and GST-IKKβ (644–756 aa) could pull down the unmodified NEMO (Fig. 6B and 6C). Strikingly, GST-CYLD (470–684 aa) could pull down neither the NEMO-SUMO-3 nor the SUMOylated NEMO (Fig. 6B, left panel and Fig. 6C), whereas GST-IKKβ (644–756 aa) could pull down both the NEMO-SUMO-3 and the SUMOylated NEMO (Fig. 6B, right panel and Fig. 6C), indicating that the SUMO-3 modification specifically impaired the interaction between NEMO and CYLD. Indeed, knockdown of SENP6 impaired the endogenous association of NEMO and CYLD (Fig. 6D). Furthermore, we found that polyubiquitination of NEMO, which activates the IKK complex, was markedly increased in the SENP6 depleted cells (Fig. 6E). However, SENP6 depletion had no influence on the expression of CYLD (Fig. 6D).

In addition, we investigated the role of the SUMO-2/3 modification of NEMO in the TLR signaling. So RNAi-resistant NEMO constructs, namely rNEMO WT or rNEMO K277A was individually employed to rescue LPS-induced NF-κB activation, in the NEMO-knockdown cells. Whereas rNEMO WT restored the induction of κB-luciferase reporter upon LPS stimuli, rNEMO K277A failed to rescue κB-luciferase induction (Fig. 6F). Taken together, these data indicate that the SUMO-2/3 modification synergizes the activation of NEMO, and SENP6 attenuates the TLR-triggered NF-κB activation via catalyzing the de-SUMOylation of NEMO.

### SENP6 is indispensable to protect mice against endotoxin shock *in vivo*

To address the *in vivo* function of SENP6 in dampening inflammation, we employed the mouse endotoxin shock model, in which mice are injected intraperitoneally with a sub-lethal dose of LPS, and then the inflammatory responses were evaluated [28].

**Figure 7:**
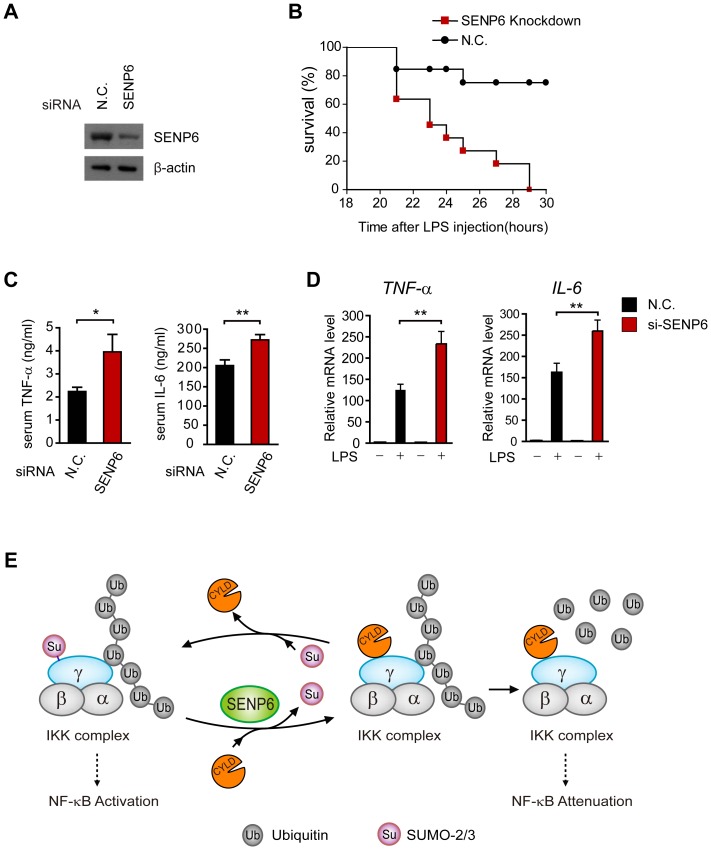
Mice deficient in SENP6 are more susceptible to LPS induced endotoxic shock. A, Immunoblot analysis of SENP6 in lysates of kupffer cells from mice at forty-eight hours after transfection with SENP6 or nonspecific (N.C.) siRNA. B, Survival of mice (n = 10 per group) transfected with SENP6 or control siRNA and forty-eight hours later injected intraperitoneally with LPS (25 mg/kg). C, ELISA of TNF-α and IL-6 in serum from mice (n = 8 per group) transfected with SENP6 or control siRNA and seventy-two hours later injected intraperitoneally with LPS (25 mg/kg), assessed two hours after LPS injection. D, Quantitative PCR of relative *TNF-α* and *IL-6* mRNA in livers from mice (n = 7 per group) transfected with SENP6 or control siRNA and seventy-two hours later injected intraperitoneally with LPS (25 mg/kg), assessed two hours after LPS injection. Data from C-D are presented as means ±S.D. from three independent experiments. *, P<0.05; **, P<0.01. E, A schematic model of the SENP6 function in TLR signaling. The deubiquitinase CYLD acts as a negative regulator of TLR signaling by interacting with NEMO and cleaving polyubiquitin chains. SUMO-2/3 could be conjugated onto NEMO, which prevents CYLD from binding to NEMO, thus strengthening the IKK activation. SENP6 reverses this process by catalyzing the de-SUMOylation of NEMO.

First, we delivered into mice, via tail vein injection, the SENP6 specific or control siRNAs coated with polyethyleneimine (PEI). The efficiency of *in vivo* ‘knockdown’ was confirmed (Fig. 7A). Next, mice were injected intraperitoneally with LPS at 25 mg/kg (the sub-lethal dose), and their survival rates were monitored. As expected, SENP6-knockdown mice were more susceptible to endotoxin shock than control mice. All the SENP6-knockdown mice died within 30 hours, whereas 80% of the control mice remained alive (Fig. 7B). Furthermore, we examined the pro-inflammatory cytokine production of the SENP6 ‘knockdown’ mice *in vivo*. Consistently, the LPS-induced cytokines (TNF-α and IL-6) were significantly elevated in the plasma of the SENP6-knockdown mice (Fig. 7C). In addition, the expression of *TNF*-α and *IL-6* mRNAs was higher in liver tissues from the SENP6-knockdown mice (Fig. 7D). Apparently, knockdown of SENP6 *in vivo* does not affect cell proliferation (Fig. S12). Collectively, these data suggest that SENP6 is indispensable for protecting mice against endotoxin shock.

## Discussion

Given the critical functions of TLR signalings in immunity, they are stringently modulated in a multi-layered and highly-ordered manner, so as to ensure that the strength and duration of the TLR signal is appropriate for any given immune response. The dynamic regulations are mainly realized by the protein post-translational modifications, in response to the intrinsic and environmental cues.

Besides phosphorylation, it is recently well established that TLR signaling molecules (TRAFs, RIPs, IRAKs, IKKs, IκBs, Rels, IRFs) are extensively regulated by ubiquitination [29], [30]. To counterbalance, A20, CYLD, OTUB1, OTUB2, Cezanne, and YopJ are implicated in promoting the de-ubiquitination of these signaling proteins [14], [31], [32], [33], [34], [35]. However, it remains a great challenge to elucidate *in vivo*, the functional relationship among these modifications, as well as the corresponding molecular mechanisms of the conformational switches. Interestingly, SUMO, the ubiquitin-like protein, is also emerging as the active modulator of the TLR signaling. For example, SUMO-1 could covalently attach itself onto TANK upon TLR7 stimulation, and this relieves the inhibition of TANK towards TLR7 signaling [36]. In addition, previous studies demonstrated that SENP1 and SENP2 could regulate the activation of IRF8 and IRF3, respectively [37], [38]. It remains to address the functions and mechanisms of the de-SUMOylation in TLR signaling. In particular, the physiological functions of other SENPs are largely unknown and this represents an evolving frontier for further investigation.

In this study, we demonstrate that SENP6 negatively regulates TLR-induced NF-κB signaling. Several lines of evidence substantiate this claim. (a) Knockdown of SENP6 results in the potentiation of the expression of the NF-κB-responsive genes induced by LPS; this effect is reversed by exogenously expressing siRNA-resistant SENP6. (b) Consistently, *in vivo* ‘knockdown’ of SENP6 induces more proinflammatory cytokines and accelerates the death rate of the mice in the endotoxic shock model. (c) The action of SENP6 is dependent on its enzymatical activity. (d) Notably, we observe that NEMO could be modified by SUMO-2/3 *in vitro and in vivo*. This modification is mapped onto the lysine residue 277 of NEMO (IKKγ). (e) Mechanistically, the SUMOylation of NEMO impairs the deubiquitinase CYLD to bind NEMO, and this prevents CYLD from removing polyubiquitin chains from NEMO, thus potentiating the IKK activation. (f) To counterbalance, SENP6 selectively interacts with the SUMOylated NEMO, but not with the unmodified NEMO. Consequently, SENP6 catalyzes the de-SUMOyaltion on the K277 of NEMO.

Since IKKα/β interact with the N-termini of NEMO, the SUMOylation of NEMO does not influence the binding affinity among IKKα/β/γ. In addition, the NEMO SUMOylation site does not overlap with its CYLD-binding domain. However, the SUMOylation effectively impairs the interaction between NEMO and CYLD, suggesting that the SUMO-moiety (∼12 kDa) might directly mask the binding surface. Another possibility is that the SUMOylation triggers a corresponding conformational change on NEMO, thus abolishing their interaction. Interestingly, SENP6 displays weak affinity towards SUMO-3 alone. We speculate that the selectivity of SENP6 to the SUMOylated NEMO may be initated by interaction bewteen SUMO-3 and SENP6, and this is further consolidated by the conformational change induced by the SUMOylation, which probably forms a new binding surface on NEMO. Future structural analysis will hopefully provide insights to the putative mechanism. Taken together, our study reveals that NEMO is modified by SUMO-2/3. This modification synergizes the polyubiquitination of NEMO and the activation of IKK kinases, via preventing the access of CYLD to the IKK complex. To counterbalance, SENP6 reverses this synergy between the SUMOylation and ubiquitination of NEMO, via removing SUMO-3 from NEMO, thus facilitating the recruitment of CYLD and attenuating the IKK activation (See Fig. 7E).

Our current model of the SENP6 action substantiates the dynamic function of the ubiquitination in regulating IKK activation, establishing a functional link between the ubiquitination and SUMOylation. SUMOylation could promote or antagonize the ubiquitination of a given substrate, depending on the topology of the modification sites. For example, the SUMOylation of HIF1α induced by hypoxia leads to its polyubiquitination and proteasome-mediated degradation, whereas IκBαmodified by SUMO-1 is resistant to ubiquitin-mediated degradation [16], [22]. To our knowledge, SENP6 represents the first case that the de-SUMOylation facilitates the binding of a specific deubiquitinase.

The SUMO modification is highly dynamic, and it is catalyzed antagonistically by SUMO E2/E3 ligases and SENPs. It is not known about the identity of the potential E3 that catalyzes the SUMO-2/3 conjugation onto NEMO. It is speculated that Ubc9 (E2) is sufficient for this conjugation. SENP6 catalyzes the deconjugation of SUMO-3 from NEMO. In contrast, SENP1, SENP2 or SENP3 did not influence NEMO SUMOylation. Neither could any of them modulate the NF-κB activation stimulated by TLRs, highlighting the functional importance of SENP6 in inflammation and immunity. Interestingly, SENP6 has a unique insertion, not found in other SENPs, that splits the conserved catalytic domain. Whether this motif functions as a substrate determinant remains to be explored by structural analysis.

SENP6 (also named SUSP1), was recently identified as the largest member (1112 amino acids) of the SUMO-specific proteases family (the C48 cysteine proteases) [39]. Little is known about the physiological functions of SENP6, although the biochemical properties of SENP6 is established. It is recently suggested that SENP6 could modulate the SUMOylation of CENP-1 and PML. However, the underlying mechanism and functional consequences need further exploration. In addition, SENP6 could potentially modulates the SUMOylation status of RPA70, a pivotal factor for DNA repair [27]. In this study, we have linked SENP6 to dynamically regulate the IKK activation. Future studies will be focused on generating the SENP6 knockout mice and analyzing its critical function in immunity and inflammation. Biophysical approach will also be employed to probe the transient ubiquitination and SUMOylation status of NEMO, linking them to the activation status of IKK and NF-κB.

## Materials and Methods

### Ethics statement

C57BL/6 mice 6–8 weeks old were purchased from the Shanghai SLAC Laboratory Animal Company. The mice were maintained under specific pathogen-free (SPF) conditions at the Shanghai Institute of Biochemistry and Cell Biology. Animal experiments were carried out in strict accordance with the regulations in the Guide for the Care and Use of Laboratory Animals issued by the Ministry of Science and Technology of the People's Republic of China. The protocol was approved by the Institutional Animal Care and Use Committee of the Shanghai Institute of Biochemistry and Cell Biology, Chinese Academy of Sciences (Permit Number: IBCB0027 Rev2).

### Cell culture and transfection

HEK293T, MEF and RAW264.7 cells were cultured using DMEM (Invitrogen) plus 10% FBS (Gibco), supplemented with 1% penicillin-streptomycin (Invitrogen). Lipofectamine 2000 (Invitrogen) was used for transient transfection of HEK293T Cells. MEF and RAW264.7 cells were transfected with Gene Pulser Xcell (Bio-Rad). Small interference RNA was transfected with Lipofectamine 2000 (Invitrogen) according to the manufacturer's instructions.

### Reagents

The polyclonal antibody against SENP6 was a gift from Dr. Ronald T. Hay (University of Dundee, U.K.). Mouse anti-SENP6 antibody was obtained from Abnova. The antibodies against hemagglutinin (HA), Myc, NEMO, CYLD and SENP3 were purchased from Santa Cruz Biotechnology. Flag, His, Ub and β-actin antibodies were obtained from Sigma-Aldrich. SUMO-2/3 antibody was purchased from Zymed. Phospho-IκBα, phospho-NF-κB p65 and phospho-JNK antibodies were from Cell Signaling. Human recombinant TNF-α was purchased from R&D Systems (Minneapolis, MN). N-ethylmaleimide (NEM), phorbol 12-myristate 13-acetate (PMA), Doxorubicin (DOX) and LPS were purchased from Sigma-Aldrich. The complete protease inhibitor cocktail and the PhosSTOP phosphatase inhibitor cocktail were obtained from Roche. The siRNA duplexes targeting SENPs and NEMO were chemically synthesized by Gene-Pharma. The sequences of siRNAs are shown as follows: m*SENP6*-1#, 5′-GGG UGA UAA AGC CUG UAA ATT-3′; m*SENP6*-2#, 5′-CAA CUA AUC UGU CGA UAC ATT-3′; m*SENP7*-1#, 5′-GGA CGA GAA UUC AGA AAG ATT-3′; m*SENP7*-2#, 5′-GCC UUA UGC UCU UAG AAA UTT-3′; h*SENP1*, 5′-GUG AAC CAC AAC UCC GTA UUC-3′; h*SENP2*, 5′-GGG AGU GAU UGU GGA AUG UTT-3′; h*SENP3*, 5′-GCU UCC GAG UGG CUU AUA ATT-3′; h*SENP5*, 5′-GUC CAC UGG UCU CUC AUU ATT-3′; h*SENP6*, 5′-GAC UUA ACA UGU UGA GCA ATT-3′; h*SENP7*, 5′-CAA AGU ACC GAG UCG AAU AUU-3′; *NEMO*, 5′-CCA UGA GUC AGC CAG GAU UTT-3′; The nonspecific siRNA (N.C.), 5′-UUC UCC GAA CGU GUC ACG UTT-3′.

### Plasmids and recombinant proteins

SENP1, SENP2, SENP3, SENP5, SENP6, SENP7, MyD88, TRAF6, p65, IKKα, IKKβ, NEMO, CYLD cDNAs were obtained by PCR from the thymus cDNA library and subsequently inserted into mammalian expression vectors as indicated. The reporter plasmids (5×κB-luciferase, E-selectin-luciferase, AP-1-luciferase and pTK-Renilla) have been described previously [40]. SUMO-2, SUMO-3 constructs were kindly provided by Dr. Jinke Cheng (School of Medicine, Shanghai Jiao Tong University, Shanghai, China). The SENP6 siRNA-resistant form was generated with silent mutations introduced into the siRNA target sequence. All point mutations were generated by using a QuickChange XL site-directed mutagenesis method (Stratagene). All the plasmids were verified by sequencing. Recombinant GST-fusion proteins were purified from Escherichia coli (BL21) by using glutathione-Sepharose 4B resin (GE Healthcare, Piscataway, NJ).

### Immunoprecipitation assay and immunoblot analysis

For immunoprecipitation assay, cells extracts were prepared by using RIPA buffer (50 mM Tris-HCl pH 7.4, 150 mM NaCl, 1 mM EDTA, 1% Triton X-100, 0.1% SDS, 0.5% deoxycholate) supplemented with a complete protease inhibitor cocktail (Roche) and 20 mM N-ethylmaleimide (NEM). Lysates were incubated with the appropriate antibody for four hours to overnight at 4°C before adding protein A/G agarose for two hours. The immunoprecipitates were washed three times with the same buffer and eluted with SDS loading buffer by boiling for five minutes.

For denaturing immunoprecipitation, cells were lysed in 1% SDS buffer (50 mM Tris-HCl pH 7.5, 150 mM NaCl, 1% SDS, 10 mM DTT) and boiled for thirty minutes. The lysates were centrifuged and diluted by 10-fold with Lysis buffer (50 mM Tris-HCl pH 7.5, 150 mM NaCl, 1 mM EDTA, 1% Triton X-100). The diluted lysates were immunoprecipitated with the indicated antibodies for four hours to overnight at 4°C before adding protein A/G agarose for two hours. After extensive wash, the immunoprecipitates were subjected to immunoblot analysis.

For immunoblot analysis, the samples were subjected to SDS-PAGE. The resolved proteins were then electrically transferred to a PVDF membrane (Millipore). Immunoblotting was probed with indicated antibodies. The protein bands were visualized by using a SuperSignal West Pico chemiluminescence ECL kit (Pierce). Signal intensities of immunoblot bands were quantified by Image J software.

### Ni-NTA pulldown analysis

For Ni-nitrilotriacetic acid resin (NTA) pulldown analysis, cells were lysed in His-Lysis Buffer (50 mM Tris-HCl pH 7.4, 300 mM NaCl, 1% Triton X-100, 20 mM imidazole, 10 mM β-ME) supplemented with 1 mM PMSF. After centrifugation, the supernatants were collected and incubated with 20 µL Ni-NTA agarose beads (Qiagen) for four hours at 4°C. The precipitates were washed three times with His-Lysis Buffer, and were boiled with SDS loading buffer, and then subjected to SDS-PAGE followed by immunoblot analysis.

### GST pulldown analysis

For GST pulldown analysis, purified recombinant GST-fusion proteins were bound to GST resin (GE Healthcare) by incubating for two hours followed by extensive washing. Cell extracts were prepared by the similar method as immunoprecipitation analysis. The preloaded GST resin were added into the cell extracts and then incubated for four hours at 4°C. Precipitates were extensively washed before loading onto SDS-PAGE. The indicated proteins were revealed by immunoblot analysis.

### Luciferase reporter assays

Luciferase reporter assays were performed as described previously [41].

### Real-time RT-PCR

Total RNA was isolated from indicated cells by using TRIzol reagent (Invitrogen) according to the manufacturer's instructions, and then subjected to reverse transcription. The quantifications of gene transcripts were performed by real-time PCR using Power SYBR GREEN PCR MASTER MIX (ABI). GAPDH served as an internal control. PCR primers used to amplify the target genes are shown as follows: *GAPDH*: sense (5′-GAA GGG CTC ATG ACC ACA GT-3′), antisense (5′-GGA TGC AGG GAT GAT GTT CT-3′); *TNF-α*: sense (5′-CAT CTT CTC AAA ATT CGA GTG ACA A-3′), antisense (5′-CCA GCT GCT CCT CCA CTT G-3′); *IL-6*: sense (5′-GAG AGG AGA CTT CAC AGA GGA TAC-3′), antisense (5′-GTA CTC CAG AAG ACC AGA GG-3′); *ICAM-1*: sense (5′-CAT CCC AGA GAA GCC TTC CTG-3′), antisense (5′-TCA GCC ACT GAG TCT CCA AGC-3′).

### Measurement of cytokines

Concentrations of the cytokine in culture supernatants were measured by ELISA kit (R&D Systems) according to the manufacturer's instructions.

### Immunostaining and confocal microscopy

Cells grown on coverslips were fixed for 15 min with 4% paraformaldehyde in PBS, permeabilized for 20 min in 0.1% Triton X-100 in PBS and blocked using 5% BSA for 1 hour. Then, the cells were stained with the indicated primary Abs followed by incubation with a Cy3-conjugated goat anti-rabbit IgG (Jackson ImmunoResearch). Nuclei were counterstained with DAPI (Sigma-Aldrich). Slides were mounted using Aqua-Poly/Mount (Polysciences, Warrington, PA). Images were captured at room temperature using a confocal microscope (TCS SP2 ACBS; Leica) with a ×63 (numerical aperture 1.4) oil objective. The acquiring software was TCS (Leica, Solms, Germany).

### *In vivo* siRNA transfection and *in vivo* endotoxic shock model

The siRNA was delivered into C57BL/6 mice with JetPEI transfection reagent (PolyPlus Transfection, San Marcos, CA) according to the manufacturer's instructions. The siRNA and JetPEI was each diluted into 100 µl of 5% glucose, then mixed and incubated for fifteen minutes at room temperature at a final N/P ratio of 8. Finally, the mixture (200 µl) was injected into each mouse via tail vein.

For the LPS-induced endotoxicity study, after seventy-two hours of siRNA delivery *in vivo*, the mice were challenged intraperitoneally with LPS at a dose of 25 mg/kg. Then, two hours later, relative mRNA in livers was measured by quantitative real-time RT-PCR. Cytokines IL-6 and TNF-α from SENP6-knockdown or control mice were also measured two hours after intraperitoneal injection of LPS (25 mg/kg) by ELISA kits (R&D Systems) according to manufacturer's instructions.

For the LPS-induced endotoxic shock study, mice were injected intraperitoneally with 25 mg/kg LPS after forty-eight hours of *in vivo* siRNA transfection. The mice were monitored for lethality for the ensuing thirty hours.

For analysis of *in vivo* ‘knockdown’ efficiency, mice were euthanized after forty-eight hours of *in vivo* siRNA transfection and then extracts of kupffer cells from livers were prepared for immunoblot analysis.

### Isolation and purification of Kupffer cells

Kupffer cells were harvested as previously reported [42], [43]. Briefly, the C57/BL6 mice were anaesthetized by intraperitoneal injection of 6 mg/ml sodium pentobarbital in saline (0.06 mg/g body weight). Then, the liver was cannulated via the portal vein, and perfused with calcium- and magnesium-free HBSS. This was followed by perfusion with HBSS containing 0.1% type IV collagenase (Worthington Biochemical Corporation). The liver was then excised and the cells dispersed in RPMI1640. The homogenate was filtered through a 70 µm cell strainer (BD Biosciences) and centrifuged at 50 g for 5 min to pellet the liver cells. Subsequently, the supernatant containing non-parenchymal liver cells was re-centrifuged at 300 g for 10 min. The cell pellet was then resuspended in 10 ml RMPI 1640, loaded onto 25% and 50% Percoll gradients, and centrifuged at 1400 g for 30 min. The cells at the interface were washed and resuspended in RMPI 1640. Kupffer cells were enriched by selective adherence to tissue culture plates.

### Cell cycle analysis

Preparations of cell suspensions for cell cycle analysis were performed as previously described [44]. Briefly, cells were fixed in ice-cold 70% ethanol at 4°C. After being washed with PBS twice, DNA was stained with 20 µg/mL propidium iodide (Sigma-Aldrich) in the presence of 200 µg/mL RNase A (Fermentas). Data were acquired on a FACSCalibur (BD Biosciences) and analyzed using CellQuest (BD Biosciences) and FlowJo (TreeStar Inc.).

### Statistics

Student's t-test was used for statistical analysis; P values of less than 0.05 were considered statistically significant.

### Accession numbers

The GenBank (http://www.ncbi.nlm.nih.gov/Genbank) accession numbers for the genes and gene products discussed in this paper are:

SENP6 (NM_146003.2, NP_666115.2), SENP7 (NM_025483.3, NP_079759.2), IKKα (NM_007700.2, NP_031726.2), IKKβ (NM_001159774.1, NP_001153246.1), NEMO (NM_001136067.2, NP_001129539.1), SUMO-2 (NM_133354.2, NP_579932.1), SUMO-3 (NM_019929.3, NP_064313.1), CYLD (NM_001128170.2, NP_001121642.1), MyD88 (NM_001172567.1, NP_001166038.1), TRAF6 (NM_009424.2, NP_033450.2), NF-κB p65 (NM_009045.4, NP_033071.1).

## Supporting Information

Figure S1 **The specific and efficient knockdown of SENPs by the corresponding siRNAs.** A–E, HEK293T cells were transfected with indicated SENPs constructs and then treated with the nonspecific control (N.C.) or indicated SENP siRNA. Cell lysates were immunoblotted with the indicated antibodies. F, HEK293T cells were transfected with siRNA as indicated. The mRNA level of *SENPs* was measured by quantitative PCR. Data in F are presented as means ± S.D. from three independent experiments. *, P<0.05; **, P<0.01.
(TIF)

Figure S2 **Knockdown of SENP7 does not affect TLR4-mediated NF-κB activation.** The indicated siRNAs were transfected into MEF cells. Induction of *IL-6* and *TNF-α* mRNA was measured by quantitative PCR after LPS (1 µg/mL) stimulation. Data are presented as means ± S.D. from three independent experiments. *, P<0.05; **, P<0.01.
(TIF)

Figure S3 **SENP6 knockdown potentiates the expression of pro-inflammatory cytokines.** MEF cells were transfected with the indicated siRNAs. After LPS (100 ng/ml), poly (I:C) (20 µg/ml) or Sendai virus stimulation, IL-6 production was determined by ELISA. Data are presented as means ± S.D. from three independent experiments. *, P<0.05; **, P<0.01.
(TIF)

Figure S4 **Loss of SENP6 has no effect on the induction of IRF3-responsive genes stimulated by LPS.** RAW264.7 cells transfected with the indicated siRNAs were stimulated with LPS (1 µg/mL) for the indicated time periods. Induction of *ISG15* and *ISG56* mRNA was measured by quantitative PCR. Data are presented as means ± S.D. from three independent experiments. n.s., not significant.
(TIF)

Figure S5 **Knockdown of SENP6 promotes poly (I:C) or Sendai virus induced NF-κB activation.** A and B, The indicated siRNAs were transfected into MEF cells. Induction of *IL-6* and *TNF-α* mRNA was measured by quantitative PCR after poly (I:C) (20 µg/ml) (A) or Sendai virus (B) stimulation. Data are presented as means ± S.D. from three independent experiments. *, P<0.05; **, P<0.01.
(TIF)

Figure S6 **SENP6 modulates TLR-triggered NF-κB activation at the IKK node.** The indicated siRNA were transfected into HEK293T cells together with 5×κB-luciferase and pTK-Renilla reporter plasmids. Forty-eight hours after transfection, cells were transfected again with MyD88, TRAF6, IKKβ and p65 for sixteen hours before luciferase assays were performed. Data are presented as means ± S.D. from three independent experiments. *, P<0.05; **, P<0.01.
(TIF)

Figure S7 **SUMO-2/3 could be covalently attached onto NEMO K277.** A, HA-IKKα, IKKβ or NEMO were individually transfected into HEK293T cells along with His-SUMO-3. Cell lysates were subjected to Ni-NTA pulldown analysis and then immunoblotted with the indicated antibodies. B, HA-NEMO was co-transfected into HEK293T along with His-SUMO-2 or His-SUMO-3. Cell lysates were subjected to Ni-NTA pulldown analysis and then immunoblotted with the indicated antibodies. C, HEK293T cells were transfected with Flag-NEMO or its mutants along with His-SUMO-3 plasmids. Cell lysates were subjected to Ni-NTA pulldown and then immunoblotted with the indicated antibodies. D, HEK293T cells were transfected with the indicated plasmids. Cell lysates were subjected to Ni-NTA pulldown and then immunoblotted with the indicated antibodies.
(TIF)

Figure S8 **Poly (I:C) or R837 promotes the SUMOylation of endogenous NEMO.** After poly (I:C) (20 µg/ml) (A) or R837 (10 µg/ml) (B) stimulation, lysates from RAW264.7 cells were immunoprecipitated with NEMO antibody or control IgG and then immunoblotted with the indicated antibodies. The intensity of the SUMOylated NEMO was quantified and normalized to that of the corresponding immunoprecipitated NEMO. The relative levels of SUMOylated NEMO are shown as fold change compared with the control.
(TIF)

Figure S9 **SENP6 modulates the status of the NEMO SUMOylation.** A, Sequence alignment of the SENPs family. Black triangle: the catalytic center of the SENP family. Black asterisk: the non-catalytic amino acid residue. B, HA-NEMO and His-SUMO-3 were transfected into HEK293T cells along with Myc-tagged SENP6, SENP6 mutants or SENP3, respectively. Cell lysates were subjected to Ni-NTA pulldown and then immunoblotted with the indicated antibodies.
(TIF)

Figure S10 **SENP6, but not SENP7, regulates the SUMOylation of NEMO.** Flag-NEMO and HA-SUMO-3 were transfected into HEK293T cells along with Myc tagged SENP6 or SENP7, respectively. Cell lysates were immunoprecipitated with Flag antibody and then immunoblotted with the indicated antibodies.
(TIF)

Figure S11 **SENP7 does not interact with NEMO.** Myc tagged SENP6 and SENP7 were individually transfected into HEK293T cells, and the cell lysates were incubated with the extracts from the HEK293T cells transfected with Flag-NEMO together with or without His-SUMO-3. The mixture was subjected to immunoprecipitation with Flag antibody and then immunoblotted with the indicated antibodies.
(TIF)

Figure S12 **Knockdown of SENP6** ***in vivo*** **does not affect immune cell cycling.** A, Representative DNA histogram of PI fluorescence in cells, as assessed by FACS. Immune cells from liver were isolated from mice transfected with the SENP6 or control siRNAs and performed cell cycle analysis. B, Ratio of cells in G0–G1 phase, S phase and G2-M phase of the cell cycle was measured by FACS and analyzed by FlowJo software. Data are presented as means ± S.D. from three independent experiments.
(TIF)
