# Supplementary figures and images for: Negative Regulation of TLR Inflammatory Signaling by the SUMO-deconjugating Enzyme SENP6
Source: PLoS Pathog. 2013 Jun 27;9(6):e1003480. doi: 10.1371/journal.ppat.1003480 (PMC3694847; doi:10.1371/journal.ppat.1003480)

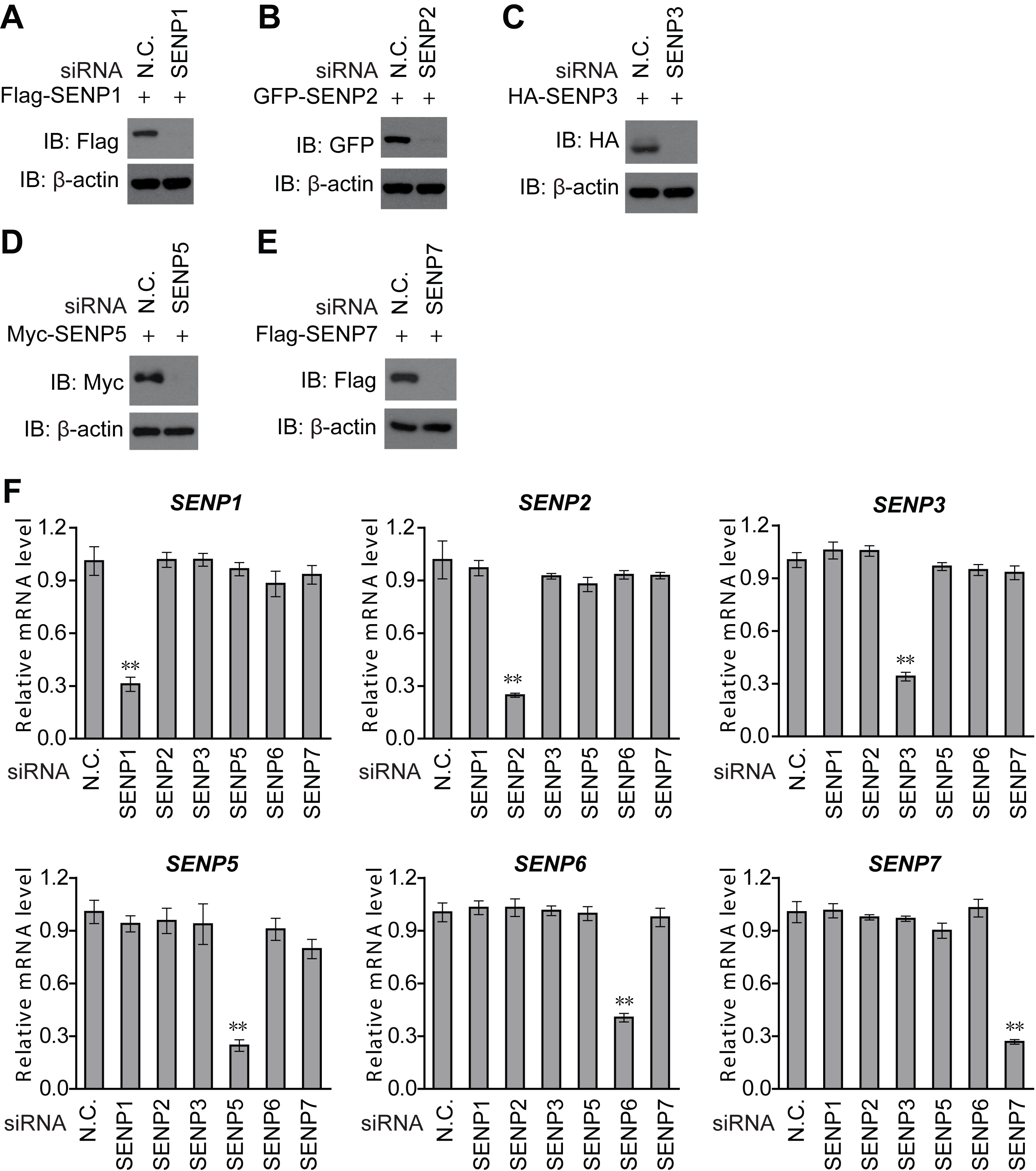

Supplement: Figure S1 — The specific and efficient knockdown of SENPs by the corresponding siRNAs. A–E, HEK293T cells were transfected with indicated SENPs constructs and then treated with the nonspecific control (N.C.) or indicated SENP siRNA. Cell lysates were immunoblotted with the indicated antibodies. F, HEK293T cells were transfected with siRNA as indicated. The mRNA level of SENPs was measured by quantitative PCR. Data in F are presented as means ± S.D. from three independent experiments. *, P<0.05; **, P<0.01. (TIF) [file ppat.1003480.s001.tif]

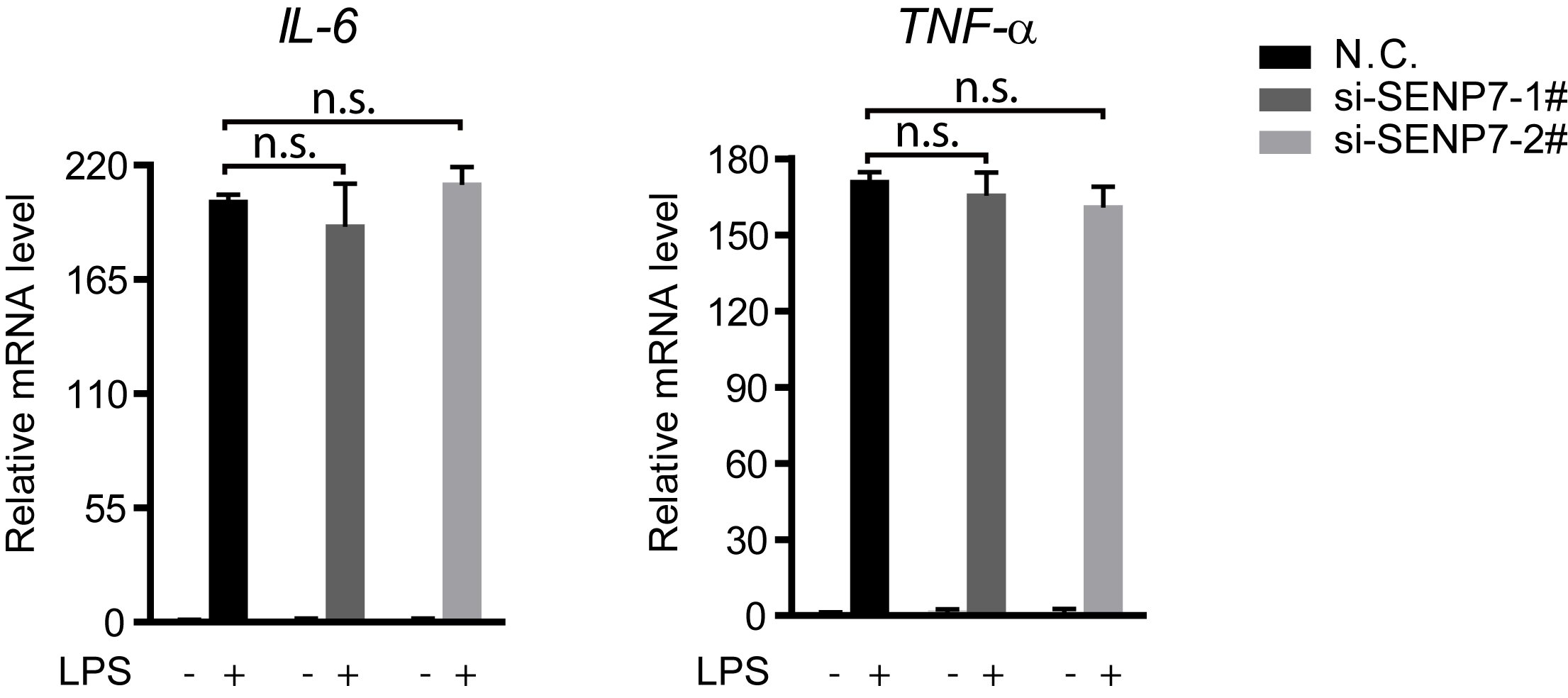

Supplement: Figure S2 — Knockdown of SENP7 does not affect TLR4-mediated NF-κB activation. The indicated siRNAs were transfected into MEF cells. Induction of IL-6 and TNF-α mRNA was measured by quantitative PCR after LPS (1 µg/mL) stimulation. Data are presented as means ± S.D. from three independent experiments. *, P<0.05; **, P<0.01. (TIF) [file ppat.1003480.s002.tif]

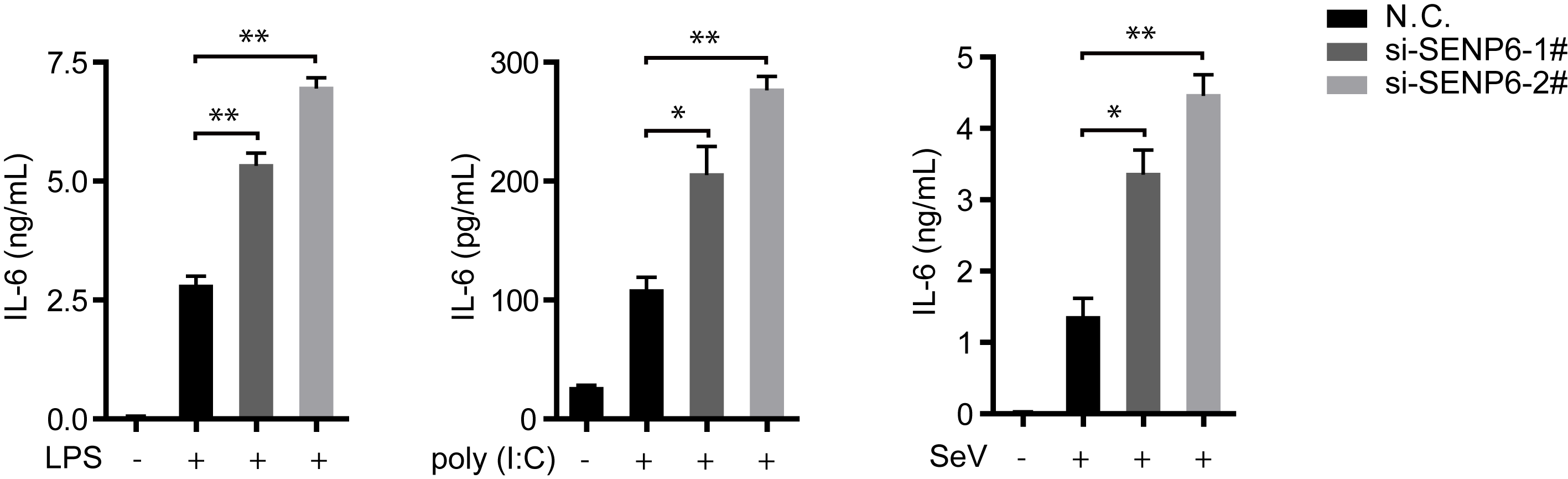

Supplement: Figure S3 — SENP6 knockdown potentiates the expression of pro-inflammatory cytokines. MEF cells were transfected with the indicated siRNAs. After LPS (100 ng/ml), poly (I:C) (20 µg/ml) or Sendai virus stimulation, IL-6 production was determined by ELISA. Data are presented as means ± S.D. from three independent experiments. *, P<0.05; **, P<0.01. (TIF) [file ppat.1003480.s003.tif]

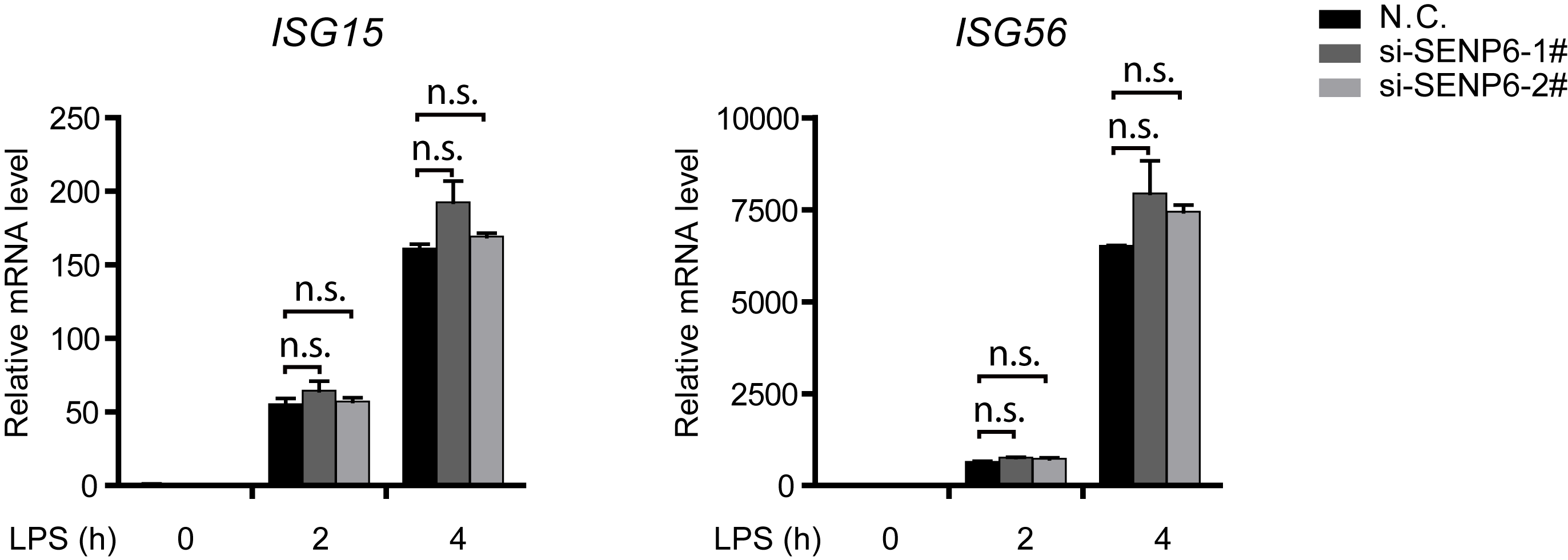

Supplement: Figure S4 — Loss of SENP6 has no effect on the induction of IRF3-responsive genes stimulated by LPS. RAW264.7 cells transfected with the indicated siRNAs were stimulated with LPS (1 µg/mL) for the indicated time periods. Induction of ISG15 and ISG56 mRNA was measured by quantitative PCR. Data are presented as means ± S.D. from three independent experiments. n.s., not significant. (TIF) [file ppat.1003480.s004.tif]

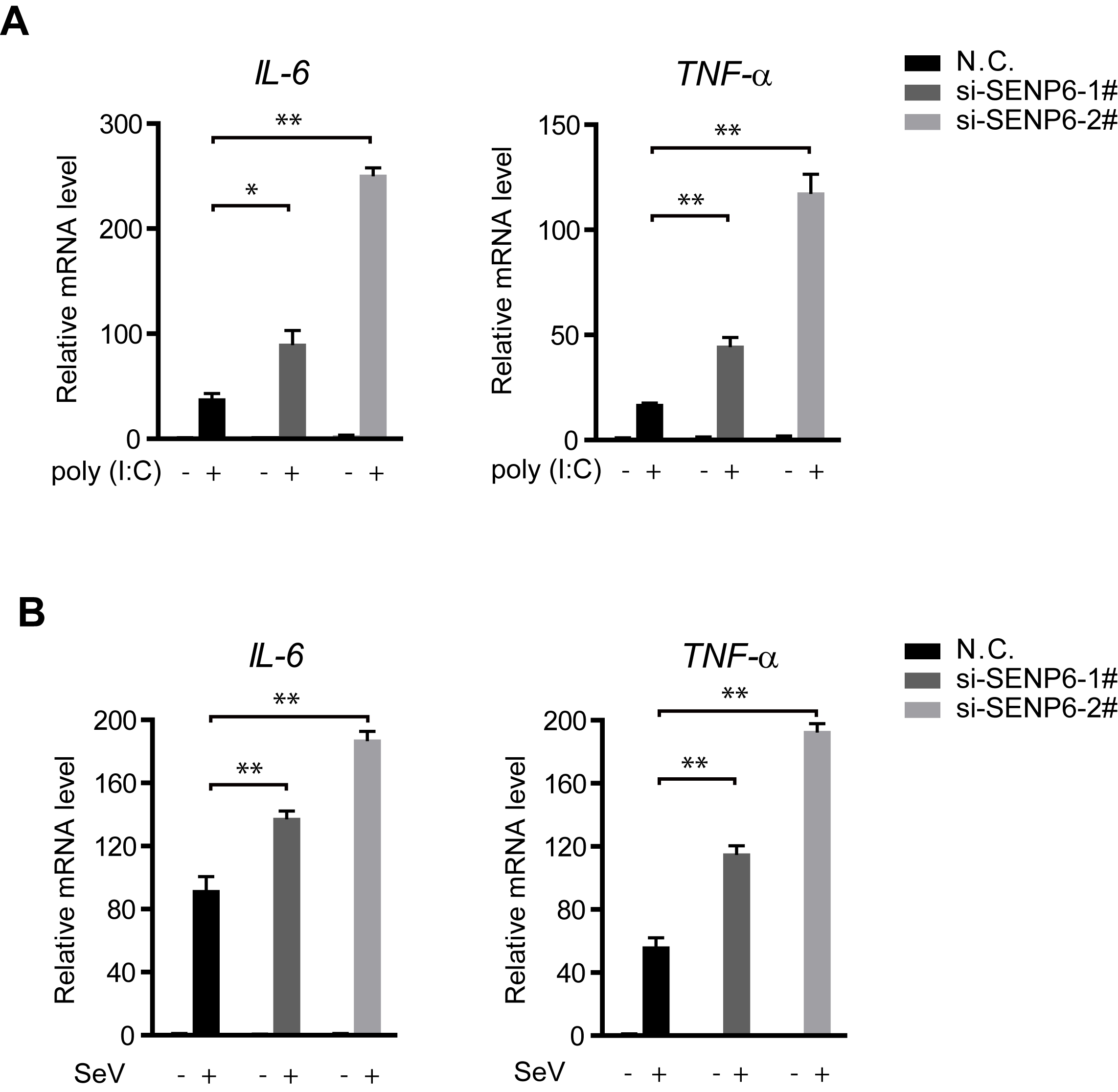

Supplement: Figure S5 — Knockdown of SENP6 promotes poly (I:C) or Sendai virus induced NF-κB activation. A and B, The indicated siRNAs were transfected into MEF cells. Induction of IL-6 and TNF-α mRNA was measured by quantitative PCR after poly (I:C) (20 µg/ml) (A) or Sendai virus (B) stimulation. Data are presented as means ± S.D. from three independent experiments. *, P<0.05; **, P<0.01. (TIF) [file ppat.1003480.s005.tif]

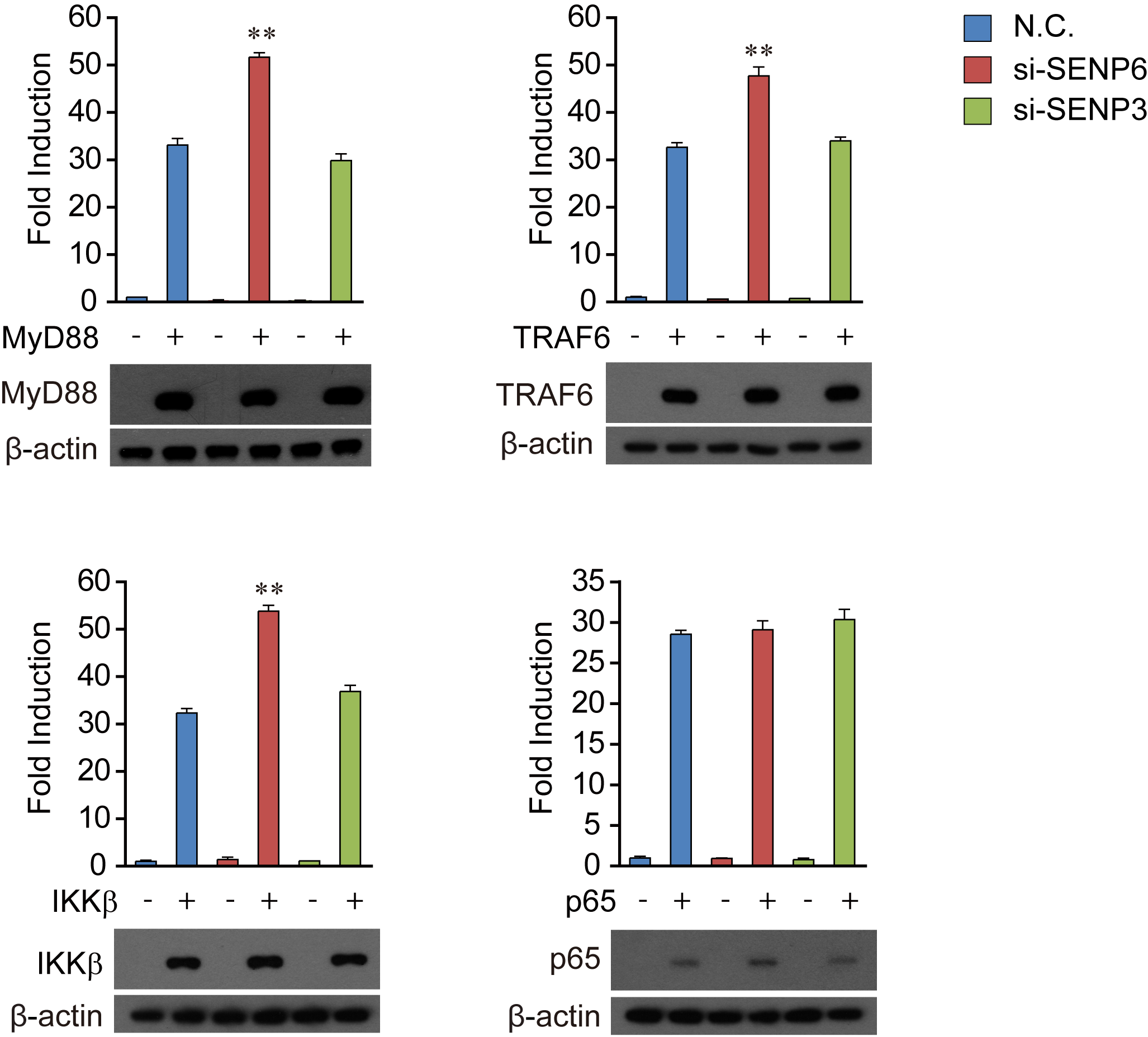

Supplement: Figure S6 — SENP6 modulates TLR-triggered NF-κB activation at the IKK node. The indicated siRNA were transfected into HEK293T cells together with 5×κB-luciferase and pTK-Renilla reporter plasmids. Forty-eight hours after transfection, cells were transfected again with MyD88, TRAF6, IKKβ and p65 for sixteen hours before luciferase assays were performed. Data are presented as means ± S.D. from three independent experiments. *, P<0.05; **, P<0.01. (TIF) [file ppat.1003480.s006.tif]

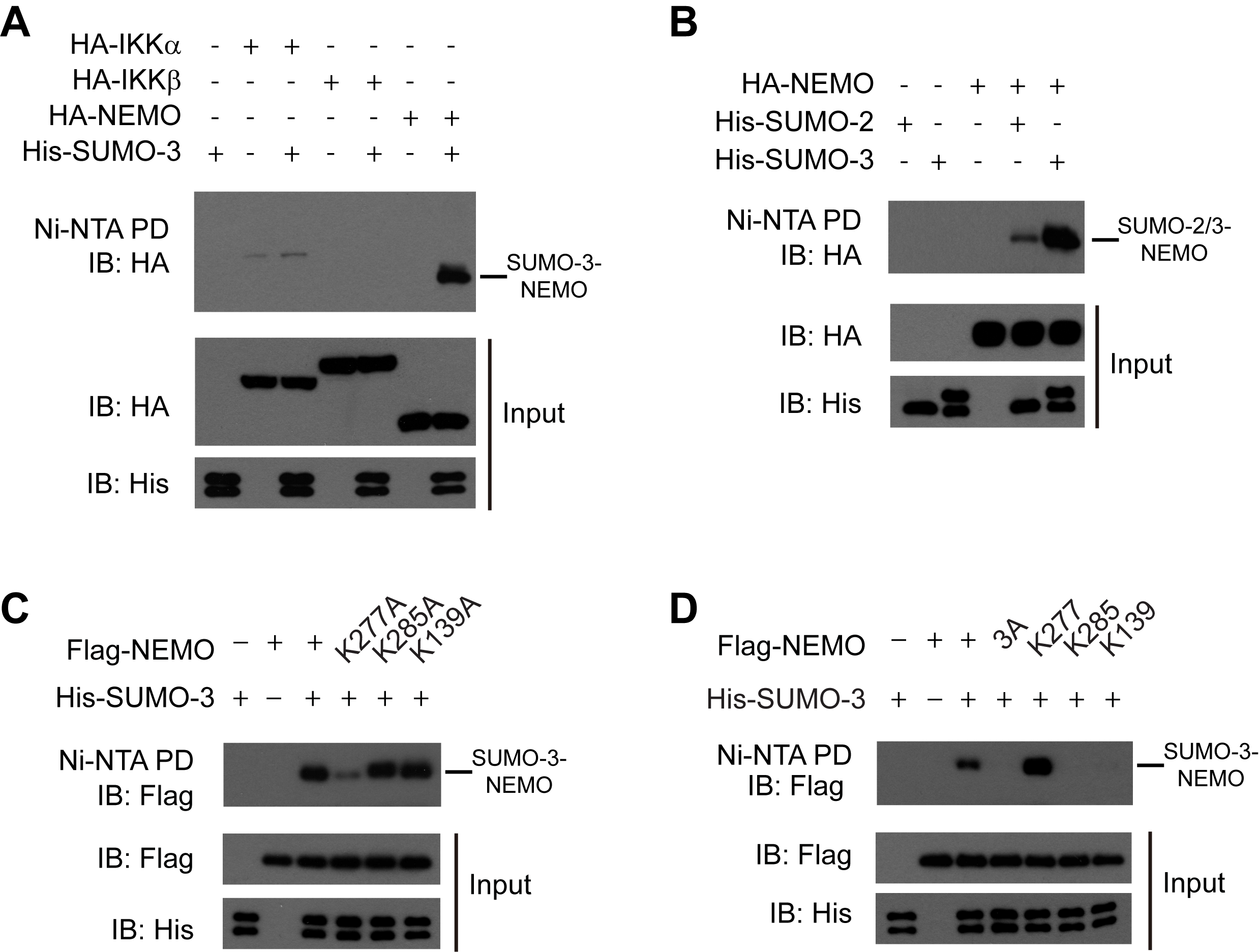

Supplement: Figure S7 — SUMO-2/3 could be covalently attached onto NEMO K277. A, HA-IKKα, IKKβ or NEMO were individually transfected into HEK293T cells along with His-SUMO-3. Cell lysates were subjected to Ni-NTA pulldown analysis and then immunoblotted with the indicated antibodies. B, HA-NEMO was co-transfected into HEK293T along with His-SUMO-2 or His-SUMO-3. Cell lysates were subjected to Ni-NTA pulldown analysis and then immunoblotted with the indicated antibodies. C, HEK293T cells were transfected with Flag-NEMO or its mutants along with His-SUMO-3 plasmids. Cell lysates were subjected to Ni-NTA pulldown and then immunoblotted with the indicated antibodies. D, HEK293T cells were transfected with the indicated plasmids. Cell lysates were subjected to Ni-NTA pulldown and then immunoblotted with the indicated antibodies. (TIF) [file ppat.1003480.s007.tif]

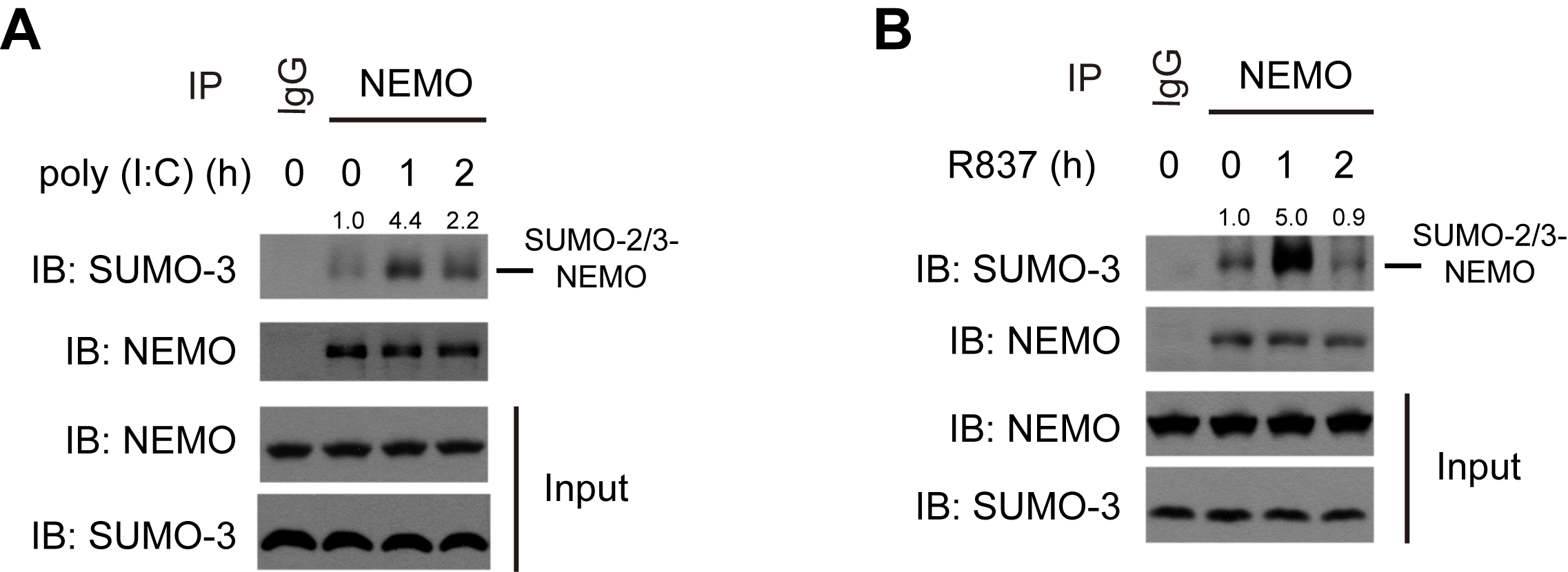

Supplement: Figure S8 — Poly (I:C) or R837 promotes the SUMOylation of endogenous NEMO. After poly (I:C) (20 µg/ml) (A) or R837 (10 µg/ml) (B) stimulation, lysates from RAW264.7 cells were immunoprecipitated with NEMO antibody or control IgG and then immunoblotted with the indicated antibodies. The intensity of the SUMOylated NEMO was quantified and normalized to that of the corresponding immunoprecipitated NEMO. The relative levels of SUMOylated NEMO are shown as fold change compared with the control. (TIF) [file ppat.1003480.s008.tif]

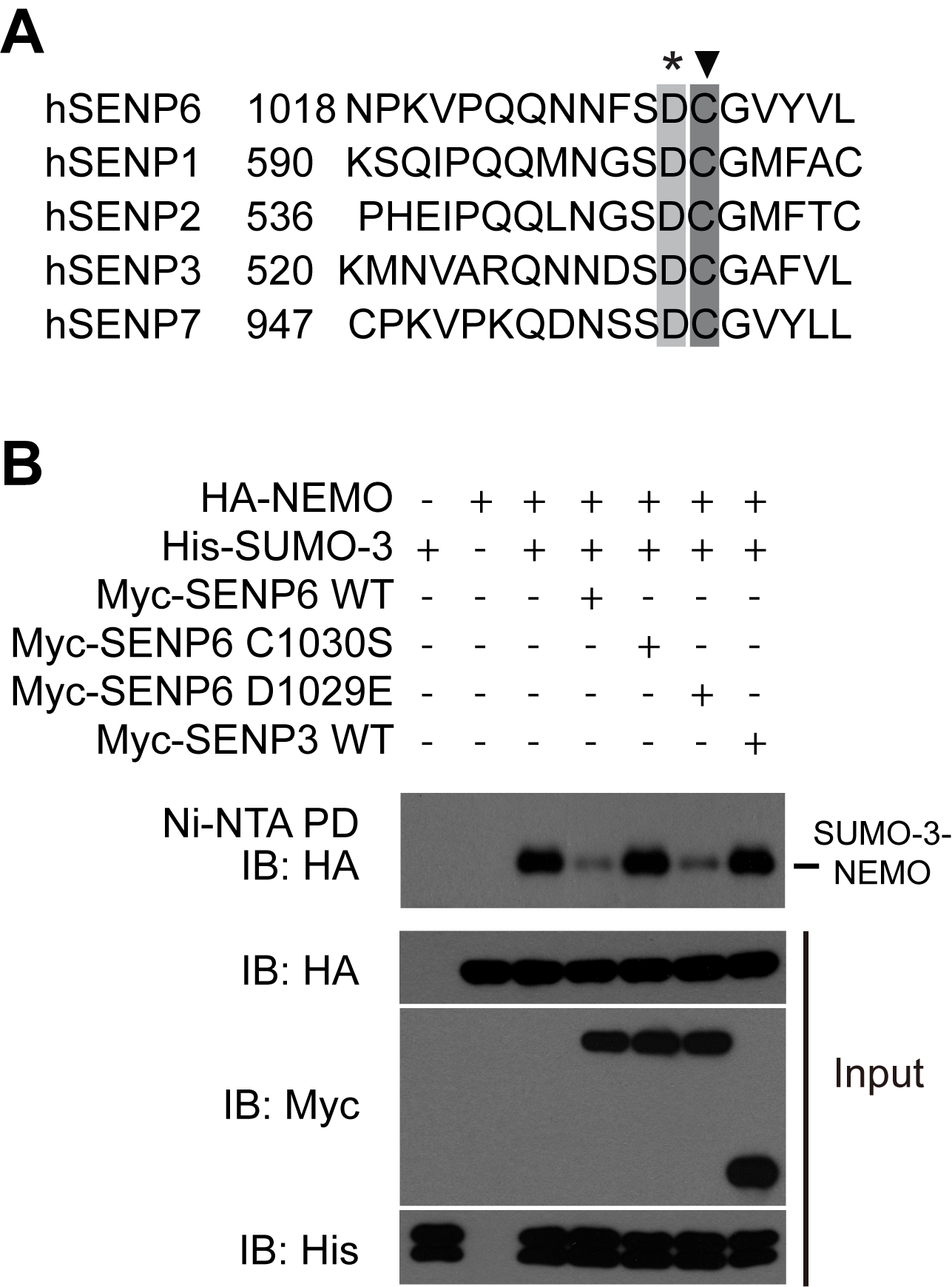

Supplement: Figure S9 — SENP6 modulates the status of the NEMO SUMOylation. A, Sequence alignment of the SENPs family. Black triangle: the catalytic center of the SENP family. Black asterisk: the non-catalytic amino acid residue. B, HA-NEMO and His-SUMO-3 were transfected into HEK293T cells along with Myc-tagged SENP6, SENP6 mutants or SENP3, respectively. Cell lysates were subjected to Ni-NTA pulldown and then immunoblotted with the indicated antibodies. (TIF) [file ppat.1003480.s009.tif]

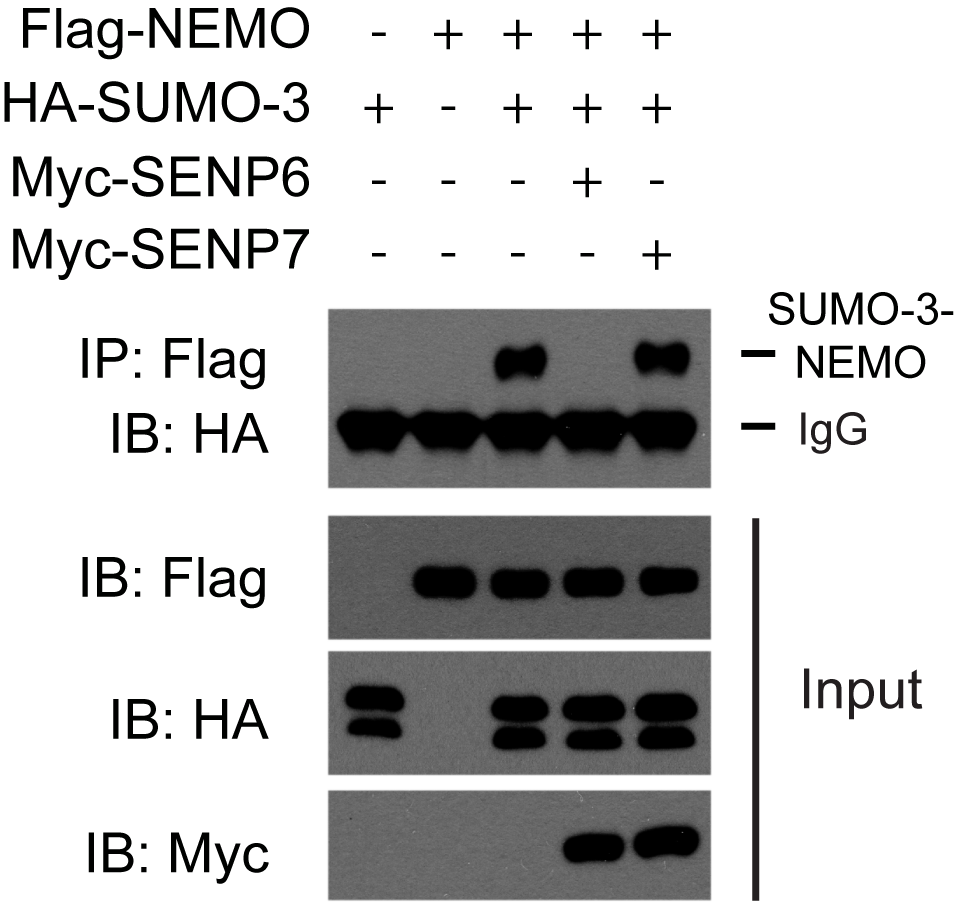

Supplement: Figure S10 — SENP6, but not SENP7, regulates the SUMOylation of NEMO. Flag-NEMO and HA-SUMO-3 were transfected into HEK293T cells along with Myc tagged SENP6 or SENP7, respectively. Cell lysates were immunoprecipitated with Flag antibody and then immunoblotted with the indicated antibodies. (TIF) [file ppat.1003480.s010.tif]

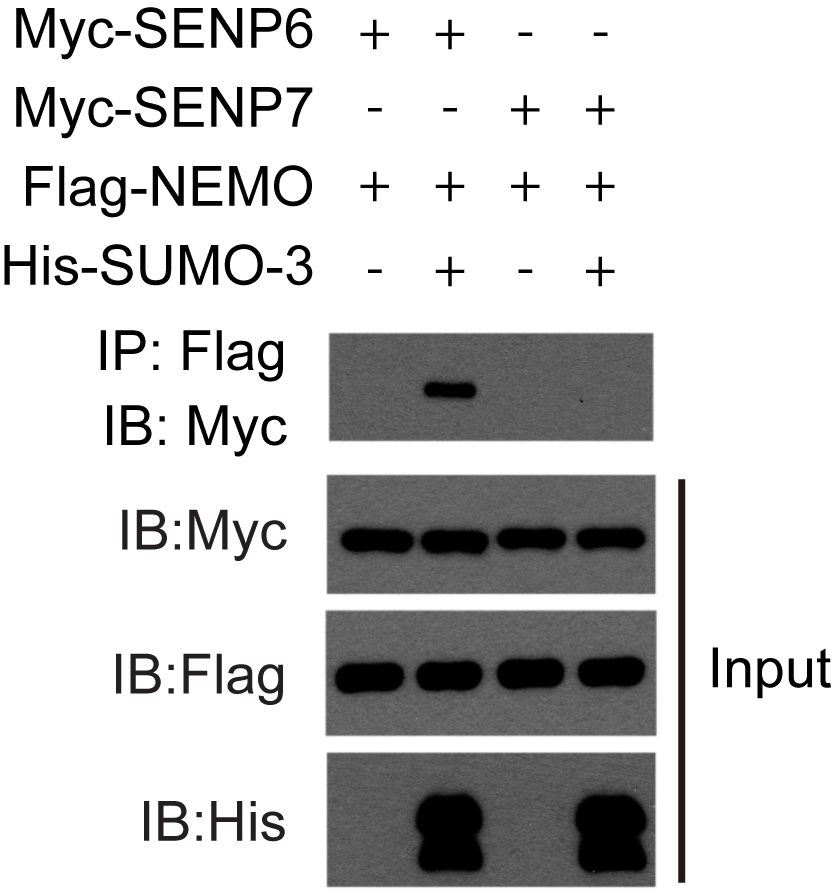

Supplement: Figure S11 — SENP7 does not interact with NEMO. Myc tagged SENP6 and SENP7 were individually transfected into HEK293T cells, and the cell lysates were incubated with the extracts from the HEK293T cells transfected with Flag-NEMO together with or without His-SUMO-3. The mixture was subjected to immunoprecipitation with Flag antibody and then immunoblotted with the indicated antibodies. (TIF) [file ppat.1003480.s011.tif]

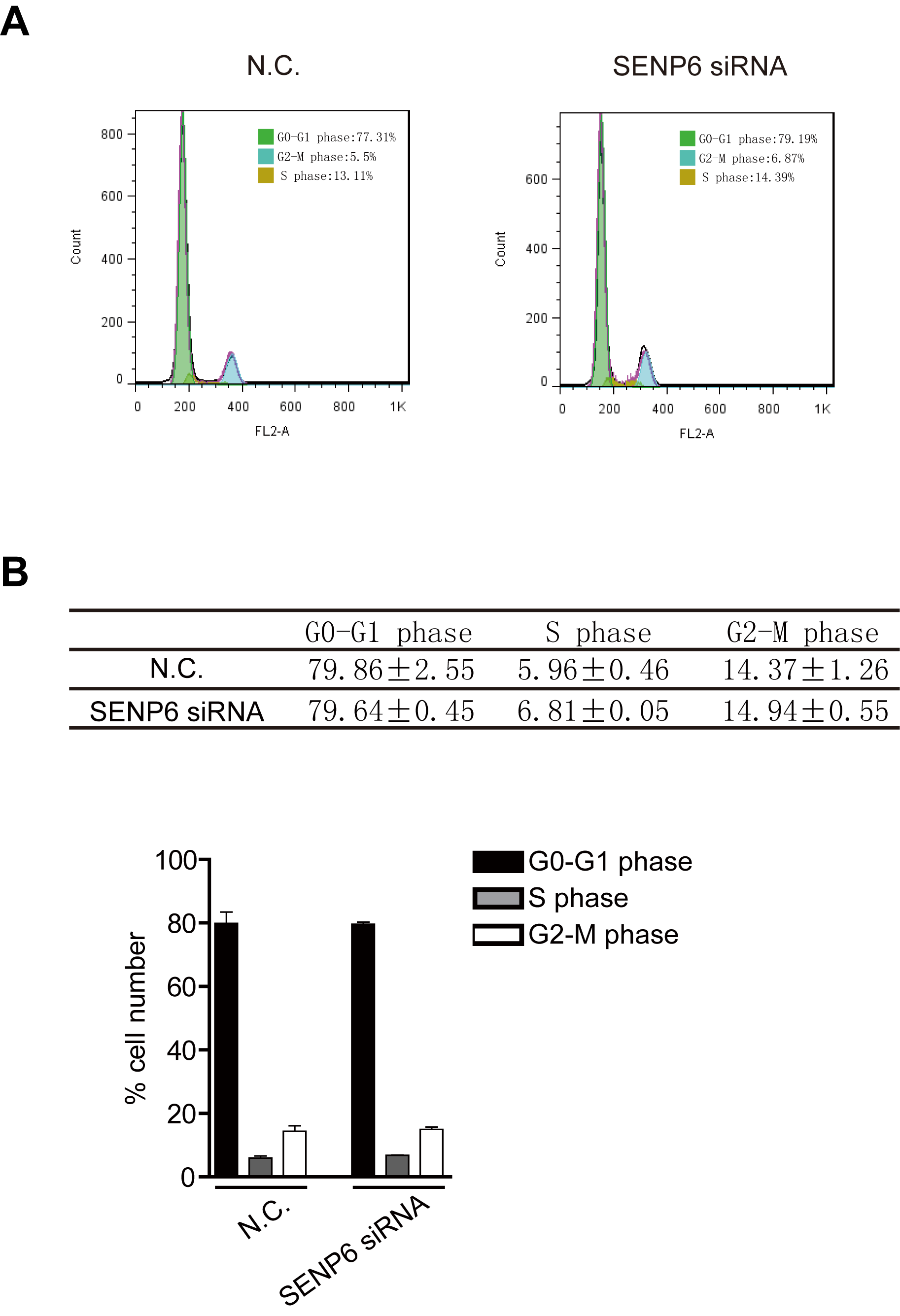

Supplement: Figure S12 — Knockdown of SENP6 in vivo does not affect immune cell cycling. A, Representative DNA histogram of PI fluorescence in cells, as assessed by FACS. Immune cells from liver were isolated from mice transfected with the SENP6 or control siRNAs and performed cell cycle analysis. B, Ratio of cells in G0–G1 phase, S phase and G2-M phase of the cell cycle was measured by FACS and analyzed by FlowJo software. Data are presented as means ± S.D. from three independent experiments. (TIF) [file ppat.1003480.s012.tif]
